# Heterologous Expression and CRISPR/Cas9-Assisted Manipulation of the Hybrid Gene Cluster Specifying the Biosynthesis of Meroterpenoids and Phenazines

**DOI:** 10.1021/acssynbio.5c00531

**Published:** 2025-12-23

**Authors:** Olha Schneider, Martin Zehl, Margherita Miele, Vittorio Pace, Corinna Brungs, Jan-Fang Cheng, Scarlet Hummelbrunner, Verena M. Dirsch, Sergey B. Zotchev

**Affiliations:** † Department of Pharmaceutical Sciences, Division of Pharmacognosy, 111090 University of Vienna, Vienna 1090, Austria; ‡ Department of Analytical Chemistry, Faculty of Chemistry, 27258 University of Vienna, Vienna 1090, Austria; § Department of Chemistry, 9314 University of Turin, Via P. Giuria 7, Turin 10125, Italy; ∥ Department of Chemistry, University of Rome “La Sapienza”, P.le A. Moro, 5, Rome 00185, Italy; ⊥ US Department of Energy Joint Genome Institute, 118576 Lawrence Berkeley National Laboratory, Berkeley, California 94720, United States

**Keywords:** Streptomyces, hybrid meroterpenoids/phenazines biosynthetic gene cluster, heterologous expression, activation, CRISPR/Cas9, marfuraquinocins

## Abstract

A hybrid gene cluster, *mfq*, predicted to govern the biosynthesis of both meroterpenoids and phenaziterpenes, was cloned from the genome of *Streptomyces* sp. S4.7 and introduced into the heterologous host *Streptomyces coelicolor* M1154. The biosynthesis of the meroterpenoids marfuraquinocins C and D, previously isolated from *Streptomyces niveus* SCSIO 3406, as well as a new congener, marfuraquinocin E, which exhibited antibacterial activity, was activated upon overexpression of the regulatory protein MfqF. However, production of neither phenaziterpenes nor phenazines was detected. The structure of marfuraquinocin E was elucidated, revealing the attachment of a terpene moiety at C-2, in contrast to C-6 as seen in the known congeners A–D. Using the CRISPR/Cas9 system, several genes in the *mfq* cluster were inactivated, confirming the role of MfqW as a prenyltransferase specific to the meroterpenoid pathway. Both gene overexpression and further knockouts provided the first insights into the complex regulation of this hybrid gene cluster. To restore the presumably deficient phenazine biosynthetic pathway, a gene encoding a PhzF homologue from another gene cluster in S4.7 was heterologously expressed alongside the *mfq* cluster, leading to the production of 1,6-phenazine dicarboxylic acid upon MfqF overexpression. This work lays the foundation for elucidating the complete biosynthetic pathway of marfuraquinocins and its potential coregulation with that of phenazines.

## Graphical abstract

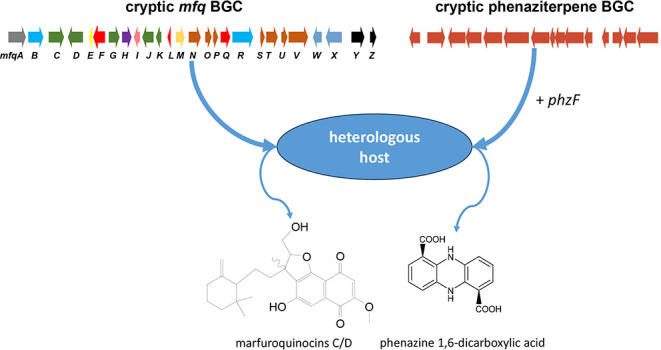

## Introduction

Bacteria that inhabit complex environments with high microbial diversity, such as soil and marine sediments, have evolved mechanisms to compete for nutrients and to communicate with other members of the microbial community by producing specialized metabolites.^1^ These molecules, known as secondary metabolites, serve as a rich source of drugs for treating human diseases, including bacterial and fungal infections (e.g., the antibiotics daptomycin and vancomycin, and the antifungal amphotericin B) and cancer (e.g., bleomycin and doxorubicin).

Secondary metabolites are biosynthesized by complex enzymatic machineries encoded by biosynthetic gene clusters (BGCs), which are assemblies of colocalized and coregulated genes that coordinate the biosynthetic process. Bacteria of the genus *Streptomyces* are among the most versatile producers of secondary metabolites, harboring up to 50 BGCs in their genomes. However, most of these clusters remain unexpressed under laboratory conditions due to the absence of yet unidentified environmental stimuli.^2^

Recent advances in genome mining aim to “awaken” transcriptionally silent BGCs using various genetic engineering-based approaches,^3^ thereby enabling the production of previously unidentified secondary metabolites and potentially advancing drug discovery programs. Nevertheless, much remains to be understood about the complex regulation of BGC expression, as not all genome mining efforts yield the expected results.

While the vast majority of characterized BGCs specify the production of a single secondary metabolite and its congeners, some complex BGCs—known as “superclusters”—encode biosynthetic pathways for chemically distinct molecules, which may exhibit synergistic biological activity.^4^ For example, a supercluster in *Streptomyces pristinaespiralis* encodes the biosynthesis of pristinamycins IA and IIA, which synergistically bind to the 50S subunit of the bacterial ribosome, thereby inhibiting protein synthesis.^5^ Another example is the *Streptomyces clavuligerus* supercluster, which specifies the biosynthesis of the cephamycins, a group of β-lactam antibiotics, and the β-lactamase inhibitor clavulanic acid.^6^ Therefore, the identification and characterization of such superclusters could facilitate the discovery and development of new drugs for treating infections and cancers that are resistant to conventional therapies.

Among the most intriguing yet incompletely characterized BGCs identified in *Streptomyces* spp. are those that appear to govern the biosynthesis of both meroterpenoids and phenaziterpenes. The naphthoquinone core of meroterpenoids, 1,3,6,8-tetrahydroxynaphthalene, is assembled by a type III polyketide synthase.^7^ This core is subsequently modified and ultimately decorated with a terpene moiety by a prenyltransferase.^8^ Meroterpenoids produced by *Streptomyces* have been reported to exhibit diverse biological activities, including antibacterial and cytotoxic properties,^9^ although their mechanisms of action remain poorly understood.

Phenazines, on the other hand, are redox-active secondary metabolites with broad-spectrum antibacterial activity, attributed to their ability to generate toxic reactive oxygen species (ROS) and disrupt cellular respiration.^10,11^ They are biosynthesized from phosphoenolpyruvate and d-erythrose 4-phosphate via the shikimate pathway, through the action of a distinct set of enzymes.^12^ In the case of phenaziterpenes, this process also involves a prenyltransferase.^13^

The first meroterpenoid/phenaziterpene “supercluster” was discovered in *Streptomyces cinnamonensis* DSM 1042,^14^ and several biosynthetic steps of these molecules were later characterized in detail.^15^ Marfuraquinocins—meroterpenoids with antibacterial and cytotoxic activities—as well as phenaziterpenes were isolated from *Streptomyces niveus* SCSIO 3406.^16^ The genome of this strain was subsequently sequenced, leading to the identification of a BGC potentially responsible for the biosynthesis of both compound classes,^7^ thus representing another example of a supercluster. However, experimental validation of this prediction has yet to be achieved.

In this study, we identified and characterized a meroterpenoid/phenaziterpene BGC in the genome of *Streptomyces* sp. S4.7, a strain isolated from the rhizosphere of Edelweiß and previously shown to produce lipopeptides known as viennamycins.^17^ We further report its heterologous expression, mutational analysis, and CRISPR/Cas9-based BGC editing, providing new insights into meroterpenoid biosynthesis and the activation of a silent phenazine pathway.

## Results and Discussion

### Cloning and Heterologous Expression of a Putative Meroterpenoid/Phenaziterpene Biosynthetic Gene Cluster

Analysis of the *Streptomyces* sp. S4.7 genome using antiSMASH 7.0 software^18^ identified a biosynthetic gene cluster (BGC) that is virtually identical to the one previously suggested to be involved in the biosynthesis of marfuraquinocins and phenaziterpenes in *S. niveus* SCSIO 3406.^7^ The organization of this BGC, along with the proposed gene functions and some of the predicted products, is shown in Figure 1.

**Figure 1:**
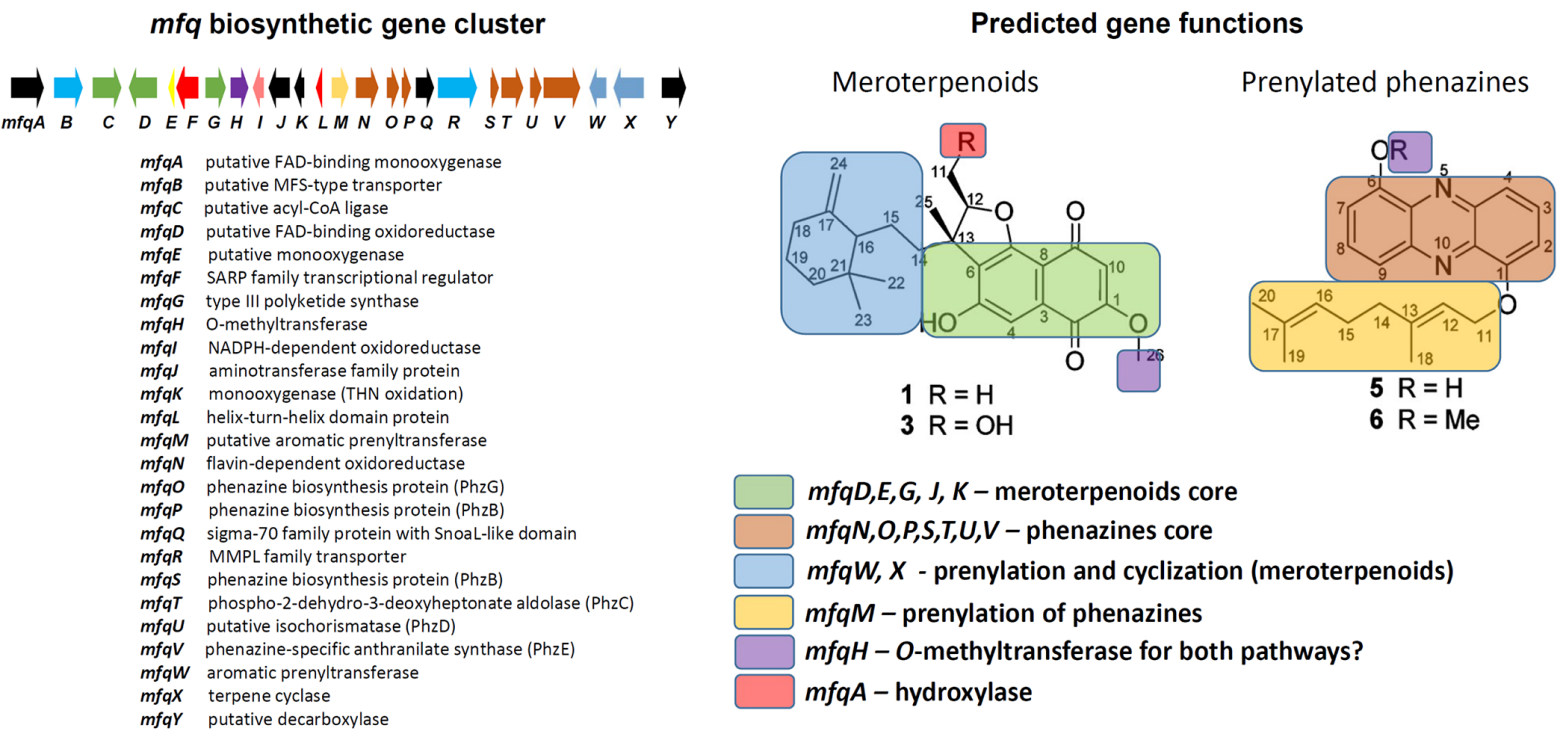
Organization of the *mfq* gene cluster with putative gene functions and its predicted products based on Song et al.^16^ and Zhu et al.^7^.

To identify and clone this BGC, provisionally named *mfq,* a genomic library of *Streptomyces* sp. S4.7 was constructed using the pCC1FOS fosmid vector and screened via pooled PCR with primers targeting the flanking regions and the center of the cluster. Fosmids yielding positive signals were end-sequenced to determine the extent of coverage of the *mfq* BGC. Overlapping inserts were then assembled into a complete *mfq* cluster using the pCLY10 vector^19^ through transformation-associated recombination in yeast (see Materials and Methods).

The recombinant plasmid harboring the *mfq* BGC was introduced into *Streptomyces coelicolor* M1154 and *Streptomyces albus* DEL14, and the resulting recombinant strains were tested for the production of marfuraquinocins and phenaziterpenes. Since neither strain produced the expected metabolites, the gene *mfqF*, encoding a *Streptomyces* Antibiotic Regulatory Protein (SARP) regulator, was cloned under the control of the strong constitutive promoter PermE* in the pUWLoriT vector^20^ and introduced into the *S. coelicolor* M1154 and *S. albus* DEL14 strains already carrying the integrated pCLY10:*mfq* plasmid.

Analysis of extracts from the fermentation of these recombinant strains revealed the production of marfuraquinocins C and/or D, which are stereoisomers that cannot be distinguished by LC-MS alone, as well as several previously unreported marfuraquinocins (Figures S2–S3, Supporting Information). However, under these conditions, no phenazine-related compounds were detected.

### Isolation and Structure Elucidation of Marfuraquinocin E

Among the previously undescribed marfuraquinocin congeners detected by LC-MS, one stood out due to its relatively high abundance, particularly in the acetone extract of the cell pellet from the *S. coelicolor* M1154 *pCLY10:mfq*/pOE_*mfqF* strain grown in MYM medium (Figures S2–S3, Supporting Information). This relatively apolar compound, named marfuraquinocin E (**1**), was isolated by silica column chromatography and preparative HPLC, followed by extensive 1D and 2D NMR spectroscopic analysis (see Materials and Methods).

High-resolution ESI-MS analysis of marfuraquinocin E revealed a molecular formula of C_26_H_34_O_5_ (HRESIMS *m*/*z* 427.2480 [M + H]^+^; calculated for C_26_H_35_O_5_^+^, *m*/*z* 427.2479, Δ = −0.2 ppm). Fragmentation of the [M + H]^+^ and [M + Na]^+^ ions by CID suggested the presence of a sesquiterpene moiety attached to a methoxylated polyketide backbone, as expected for a marfuraquinocin congener (Figures S4–S5, Supporting Information).

NMR spectroscopy identified all 26 carbon signals, 32 nonexchangeable hydrogen atoms, and one slowly exchanging proton, while two rapidly exchanging hydrogens were not observed (Table 1 and Figures S6–S12, Supporting Information). Interpretation of the 2D NMR spectra together with comparison to previously reported data for marfuraquinocin A (Table 1 and Figure 2), confirmed the presence of a 1,1-dimethyl-3-methylenecyclohex-2-yl group in the sesquiterpene side chain and a methoxy group on the polyketide moiety. However, the data were not consistent with the presence of a 1,2-dimethylfuran and the naphthoquinone substructure found in the four previously described marfuraquinocin congeners.^16^

**Table 1:** ^1^H (600 MHz, CDCl_3_) and ^13^C NMR Data (151 MHz, CDCl_3_) of Marfuraquinocin E (**1**) in Comparison with Literature Data for Marfuraquinocin A (*δ* in ppm)

	Marfuraquinocin E (**1**)		Marfuraquinocin A^a^	
Position	δ_H_ (*J* in Hz)	δ_C_, type	δ_H_ (*J* in Hz)	δ_C_, type
1	—	175.1, C	—	159.3, C
2	—	74.3, C	—	180.2, C
3	—	147.3, C	—	132.9, C
4	6.68, s	105.2, CH	7.20, s	109.5, CH
5	—	161.7, C	—	156.9, C
6	6.31, s	102.8, CH	—	129.0, C
7	—	163.8, C	—	161.1, C
8	—	109.0, C	—	109.4, C
9	—	189.7, C	—	183.6, C
10	5.54, s	101.1, CH	6.03, s	111.3, CH
11a	2.79, dd (12.3, 8.9)	44.1, CH_2_	1.45, d (6.5)	15.6, CH_3_
11b	2.53, dd (12.1, 6.8)			
12	4.56, t (7.6)	115.9, CH	4.85, q (6.5)	88.4, CH
13	—	142.0, C	—	46.9, C
14a	1.76, m	38.5, CH_2_	1.94, td (13.5, 4.5)	36.9, CH_2_
14b	1.56^b^, m		1.38, m	—
15a	1.33^b^, m	25.2, CH_2_	1.51, m	21.4, CH_2_
15b	1.20^b^, m		1.21, m	—
16	1.54, dd (11.4, 2.9)	53.9, CH	1.65, dd (11.0, 3.0)	54.8, CH
17	—	149.5, C	—	149.2, C
18	1.94, m	32.6, CH_2_	1.99, m	32.2, CH_2_
19	1.50, m	23.9, CH_2_	1.50, m	23.6, CH_2_
20a	1.42, m	36.4, CH_2_	1.36, m	36.0, CH_2_
20b	1.17, m	—	1.16, m	—
21	—	35.0, C	—	35.0, C
22	0.85, s	28.6, CH_3_	0.88, s	28.3, CH_3_
23	0.77, s	26.5, CH_3_	0.77, s	26.6, CH_3_
24a	4.68, s	109.1, CH_2_	4.73, s	109.3, CH_2_
24b	4.40, d (1.7)	—	4.55, d (2.5)	—
25	1.35, s	16.2, CH_3_	1.26, s	19.7, CH_3_
26	3.83, s	56.6, CH_3_	3.84, s	56.2, CH_3_

a Values from Song et al. (2013).

b Value determined from the HSQC spectrum.

**Figure 2:**
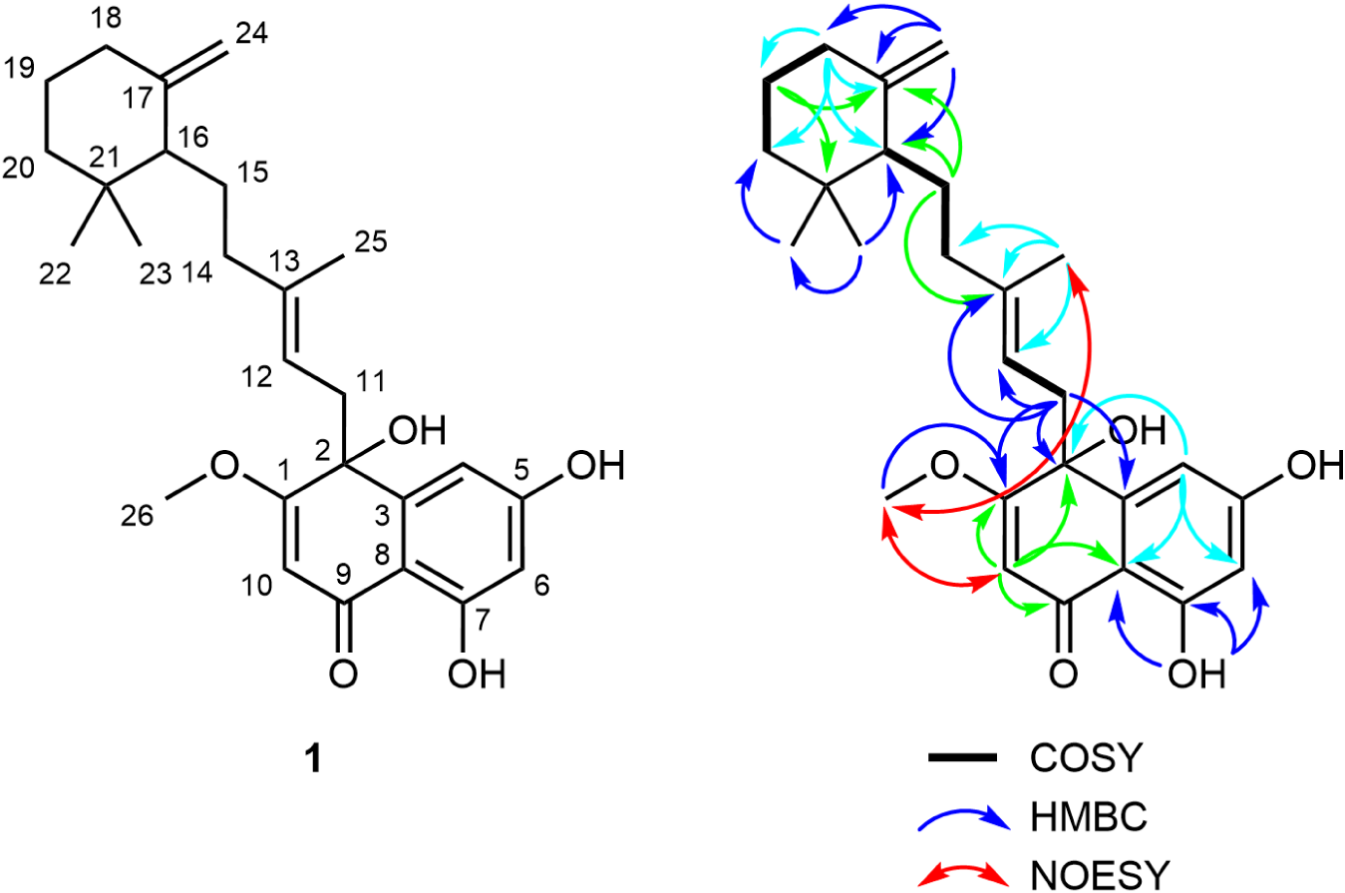
Structure of marfuraquinocin E (**1**) with atom numbering and key ^1^H–^1^H COSY, ^1^H–^13^C HMBC and ^1^H–^1^H NOESY correlations.

In marfuraquinocin E, a hydrogen is attached to C-6 instead of the sesquiterpenyl side chain, and a double bond is present between C-12 and C-13 of the latter (Figure 2). HMBC correlations from H-11 to C-1, C-2, and C-3 established that the farnesyl-derived moiety is attached to C-2 of the polyketide core, which is reduced to a naphthalenone. This interpretation is further supported by a NOESY correlation between the methoxy group and the C-25 methyl group of the side chain, as well as the strong similarity between the ^1^H and ^13^C NMR data of the naphthalenone portion of marfuraquinocin E and those of perenniporide B, a previously reported fungal natural product.^21^ The configuration at the two stereocenters C-2 and C-16 could not be determined from the NMR data.

### Biological Activity of Marfuraquinocin E

The antimicrobial activity of marfuraquinocin E was evaluated against *E. coli* DH5αF’, *P. putida* KT2440, *M. luteus* DSMZ 1790, *B. subtilis* DSMZ 10, and *S. cerevisiae* BY4742. Among the tested strains, only the *M. luteus* plates exhibited a clear inhibition zone of approximately 3 cm (Figure S13, Supporting Information).

To further investigate its activity against *M. luteus*, marfuraquinocin E was tested at concentrations ranging from 0 to 60 μg/mL in liquid TSB medium. The results indicated a minimum inhibitory concentration (MIC) of 10 μg/mL after 18 h of growth (Figure S14, Supporting Information).

Marfuraquinocin E also exhibited a concentration-dependent cytotoxic effect in human HCT116 and HepG2 cells. At a concentration of 100 μM, a significant reduction in cell viability was observed in HepG2 cells as shown by a markedly diminished fluorescence in the resazurin assay, indicating impaired metabolic activity. In the crystal violet biomass staining, next to HepG2 also HCT116 cells revealed a notable decrease in cell number or adherence in response to 100 μM marfuraquinocin E (Figure S15, Supporting Information).

These findings suggest that marfuraquinocin E exhibits potent cytotoxicity at higher concentrations in both cancer cell lines. In contrast, no significant cytotoxic effects were observed in primary HUVEC cells under the same conditions (Figure S15), suggesting potential cell-type specificity in the compound’s toxicity.

### The *Streptomyces* Antibiotic Regulatory Protein MfqF Is the BGC’s Main Regulator

To study the functionality of genes within the cluster, we employed the CRISPR/Cas9 gene-editing system, originally developed for gene editing in *S. cerevisiae* TC3.^22^ In this study, we used the same strain and system to modify the *mfq* cluster (Table 2). First, we performed experiments to obtain insights into the regulation of the *mfq* gene cluster. The cluster contains three genes that potentially encode regulatory proteins, namely *mfqF*, predicted as SARP family transcriptional regulator, *mfqL*, whose product is predicted to contain a DNA-binding helix-turn-helix domain frequently found in transcription regulatory proteins, and finally *mfqQ*, encoding a putative σ^70^ factor with a SnoaL-like domain.

**Table 2:** Brief Description of Recombinant *Streptomyces coelicolor* M1154 Strains, Their Genetic Modifications, and the Products Detected by MS after Fermenting in MYM Medium for 7 Days at 28 °C^a^

Strain	Inactivation in BGC				Expression on pUWLoriT plasmid				Products detected by LC/MS: marfuraquinocin pathway			Products detected by LC/MS: phenazine pathway	
	*mfqM*	*mfqH*	*mfqQ*	*mfqW*	*mfqF*	*mfqL*	*mfqQ*	*phzF*	Marfuraquinocins	Flaviolin	8-amino-flaviolin	Phenazi-terpenes	phenazine 1,6-dicarboxylic acid
1	-	-	-	-	-	-	-	-	-	-	-	-	-
2	-	-	-	-	**+**	-	-	-	YES	YES	YES	-	-
3	-	-	-	-	-	**+**	-	-	-	-		-	-
4	-	-	-	-	-	-	**+**	-	-	-	-	-	-
5	-	-	**+**	-	-	-	-	-	-	-		-	-
6	-	-	-	-	-	-	-	**+**	-	-	-	-	-
7	-	-	-	-	**+**	-	-	**+**	YES	YES	-	-	YES
8	-	-	**+**	-	**+**	-	-	**+**	YES	YES	-	-	-
9	-	-	**+**	-	-	**+**	-	**+**	-	-	-	-	-
10	**+**	-	-	-	-	-	-	-	-	-	-	-	-
11	**+**	-	-	-	**+**	-	-	-	YES	YES	-	-	-
12	-	-	-	**+**	-	-	-	-	-	-		-	-
13	-	-	-	**+**	**+**	-	-	-	-	YES	YES	-	-
14	-	-	-	**+**	**+**	-	-	**+**	-	YES	YES	-	YES
15	-	**+**	-	-	-	-	-	-	-	-	-	-	-
16	-	**+**	-	-	**+**	-	-	-	-	YES	YES	-	-

a Inactivations of *mfqH*, *mfqQ*, and *mfqM* and overexpression of *mfqM* and *mfqS* were performed via CRISPR/Cas9-assisted manipulation of the *Mfq* BGC. The overexpression of genes *mfqF*, *mfqL*, *mfqQ*, and *phzF* was done using a multicopy plasmid introduced into the *S. coelicolor* harbouring native or manipulated *Mfq* BGC.

As already mentioned earlier, just introducing the recombinant plasmid harboring the *mfq* BGC into *S. coelicolor* M1154 did not result in the production of any related secondary metabolites. Thus, the three genes *mfqF*, *mfqL*, and *mfqQ*, were individually expressed under the control of the *PermE* promoter on the pUWLoriT plasmid in the *S. coelicolor* strain harboring *pCLY10:mfq*. However, only overexpression of *mfqF* activated the production of compounds related to the marfuraquinocin pathway, while none of the three recombinant strains yielded phenazines (see strains 1–4 in Table 2). Next, we assessed the function of *mfqL* and *mfqQ* through deletion experiments. Multiple attempts to delete *mfqL* using the CRISPR/Cas9 system were unsuccessful, making it impossible to determine its potential regulatory role. We could, however, successfully delete *mfqQ*, but without achieving activation of the cluster (strain 5 in Table 2). These results suggest that *mfqQ* does not play a role in regulating the marfuraquinocin pathway.

### mfqW Catalyzes the Prenylation in the Marfuraquinocin Biosynthesis

Next, we wanted to confirm MfqH as the enzyme being responsible for the *O*-methylation of the marfuraquinocins and to test which of the two putative aromatic prenyltransferases MfqM and MfqW are involved in the marfuraquinocin pathway. To that end, the three respective genes were inactivated via in-frame deletion from the *mfq* cluster. The effect of these deletions on the production of the related secondary metabolites were monitored by LC-MS in both, the *S. coelicolor* strain harboring only the modified *pCLY10:mfq* vectors (as a control) and the strain additionally overexpressing the SARP regulator *mfqF*.

Deletion of *mfqH* from the heterologously expressed *mfq* cluster along with *mfqF* overexpression completely abolished the production of marfuraquinocin congeners, while two other major products were detected (strain 16 in Table 2 and Figures S16–S18). HRESIMS, MS/MS, and UV data for the main peak matched to flaviolin. Flaviolin has been previously described as biosynthetic intermediate of meroterpenoids,^8^ but according to a very recent study is more likely a shunt product resulting from spontaneous oxidation of other unstable intermediates such as 8-diazoflaviolin and 2,4,5,7,8-pentahydroxynaphthalene-1-diazonium (Figure 3).^23^ The second most abundant new metabolite is likely a biflaviolin isomer. Three isomers of biflaviolin, biosynthesized by cytochrome P450-catalyzed oxidative C–C coupling of flaviolin, have been reported in *S. coelicolor* A3(2), where they act as UV-protective pigments.^24^ The above results suggest that 2-*O*-methylation of the intermediate 1,2,4,5,7-pentahydroxynaphthalene (PHN) is an essential prerequisite for all subsequent biosynthetic steps, which cannot proceed to yield nonmethylated marfuraquinocin congeners.

**Figure 3:**
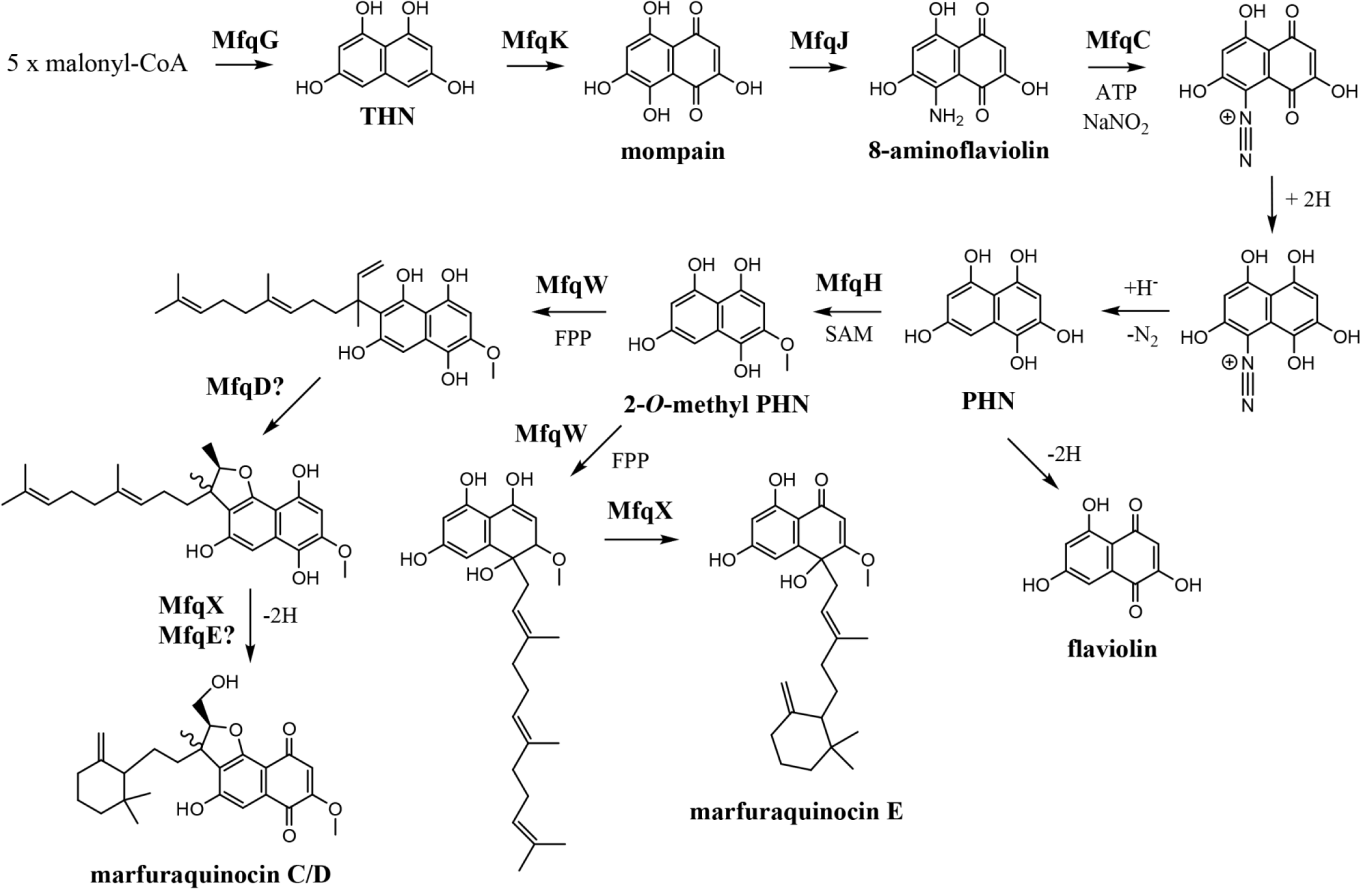
Proposed marfuraquinocins biosynthesis pathway specified by the *mfq* gene cluster in *Streptomyces* sp. S4.7. THN—tetrahydrohynaphthalene; PHN—pentahydrohynaphthalene; FPP—farnesylpyrophosphate; SAM—*S*-adenosylmethionine.

Inactivation of *mfqM*, which encodes one of two aromatic prenyltransferases, did not result in significant changes in meroterpenoid production, either in the presence or absence of SARP expression (strain 10 and 11 in Table 2 and Figure S18). This indicates that MfqM may be specific to the phenaziterpene biosynthesis.

The *mfqW* gene, encoding a putative aromatic prenyltransferase, was inactivated using the same procedure as for *mfqM*. LC-MS analysis of extracts from the recombinant strain harboring pCLY10:*mfq*::Δ*mfqW* and overexpressing *mfqF* revealed accumulation of oxidized marfuraquinocin precursors, specifically 2-*O*-methylflaviolin and flaviolin (strains 12 and 13 in Table 2 and Figure S19).

These data strongly suggest that MfqW functions as the prenyltransferase responsible for attaching a farnesyl moiety to 2-*O*-methylflaviolin (Figure 3). In order to confirm the MfqW function, a codon optimized version of *mfqW* was expressed in *Escherichia coli*, purified, and activity of recombinant protein tested with 2-*O*-methylflaviolin, synthesized as described in Supporting Information (Method S1), and FPP, DMAPP or GPP (see Materials and Methods). However, no prenylation of the 2-*O*-methylflaviolin with either of the prenyl donors was observed under the conditions tested. Our original biosynthetic hypothesis, based on previously proposed meroterpenoid pathways,^8,14,15^ was that MfqW acts on the oxidized naphthoquinone form, while more recent data suggest that the physiological substrate is the reduced form, 2-O-methyl-1,2,4,5,7-pentahydroxynaphthalene (PHN).^23^ Hence, new enzyme assays were performed with both FPP, DMAPP and GPP and 2-*O*-methylflaviolin reduced to PHN by dithionite, as described in Noguchi et al.^23^ Surprisingly, only geranyl-PHN was detected in these experiments (Figure S22, Supporting Information), which contradicted the in vivo data for the *mfqW* deletion mutant. Nevertheless, despite the lack of observable farnesylation in vitro, we propose that FPP is the physiological prenyl donor in vivo, consistent with the structure of the isolated marfuraquinocins and the accumulation of precursor observed in the Δ*mfqW* mutant. The discrepancy between the in vivo and in vitro donor preferences of MfqW likely arises from differences in substrate form, assay conditions, and enzyme environment. While MfqW catalyzes geranylation of the PHN in vitro, genetic and metabolomic evidence indicate that it functions as a farnesyltransferase in vivo. Intracellular metabolite pools in bacteria can determine the preferred prenyl donor. For example, DMAPP and FPP levels are generally high in bacteria, because they feed essential pathways for sterols and quinones, while GPP is often scarce, especially in bacteria that lack a cis-GPP synthase.^25^

Based on these findings and prior knowledge of meroterpenoid biosynthesis,^8,23,26,27^ a plausible biosynthetic pathway leading to marfuraquinocins C/D and the newly identified congener marfuraquinocin E is proposed (Figure 3). Verification of other specific steps in this pathway, which currently remain largely hypothetical, and characterization of the associated enzymes are ongoing.

### Activation of the Silent Phenazine Biosynthesis Pathway

None of the gene modifications described above led to the detection of any phenazines, suggesting that the *mfq* genes presumably involved in phenazine biosynthesis (Figure 1) were either not expressed under the tested conditions or that the biosynthetic pathway was incomplete.

The biosynthesis of the phenazine core, culminating in phenazine-1,6-dicarboxylic acid (PDC), is relatively well understood in bacteria.^28^ A closer examination of the *mfq* genes presumed to participate in phenaziterpene biosynthesis revealed that they encode most, but not all, of the enzymes required for the formation of the phenazine core structure up to PDC (Figure 4A). Notably, the gene encoding trans-2,3-dihydro-3-hydroxyanthranilate isomerase (a PhzF homologue), which catalyzes the isomerization of (2*S*,3*S*)-2,3-dihydro-3-hydroxyanthranilic acid to (1*R*,6*S*)-6-amino-5-oxocyclohex-2-ene-1-carboxylic acid, was absent from the *mfq* cluster. The lack of the enzyme responsible for this key step in phenazine biosynthesis likely explains why we could not observe any phenazines in the recombinant strains so far.^29^

**Figure 4:**
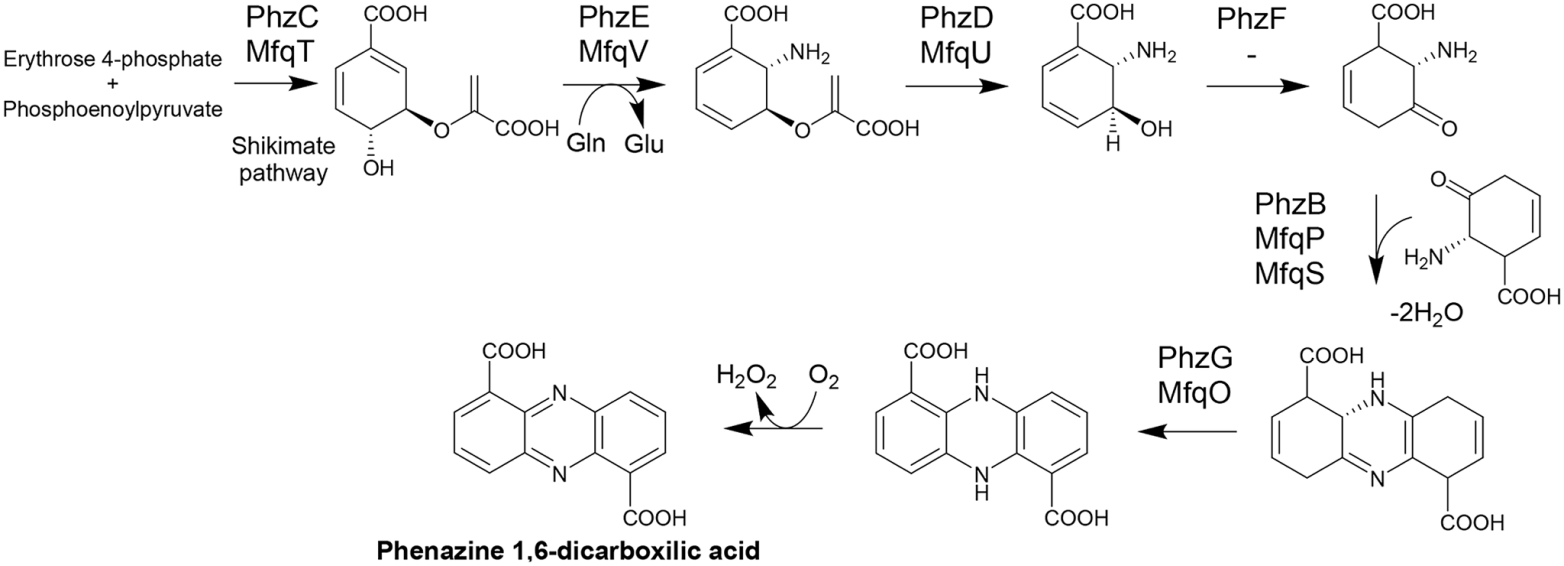
Initial steps in the phenazines biosynthesis in bacteria according to Huang et al.,^28^ and corresponding Mfq proteins encoded within the *mfq* gene cluster.

However, the genome of *Streptomyces* sp. S4.7 contains another gene cluster predicted to be involved in the biosynthesis of prenylated phenazines, but it includes only four genes required for phenazine core formation: homologues of *phzB*, *phzC*, *phzD*, and *phzF* (Figure 5B). Thus, it seemed plausible that this secondary BGC and the *mfq* cluster may cross-complement each other in phenazine core biosynthesis, although the predicted cognate prenyltransferases are highly dissimilar.

**Figure 5:**
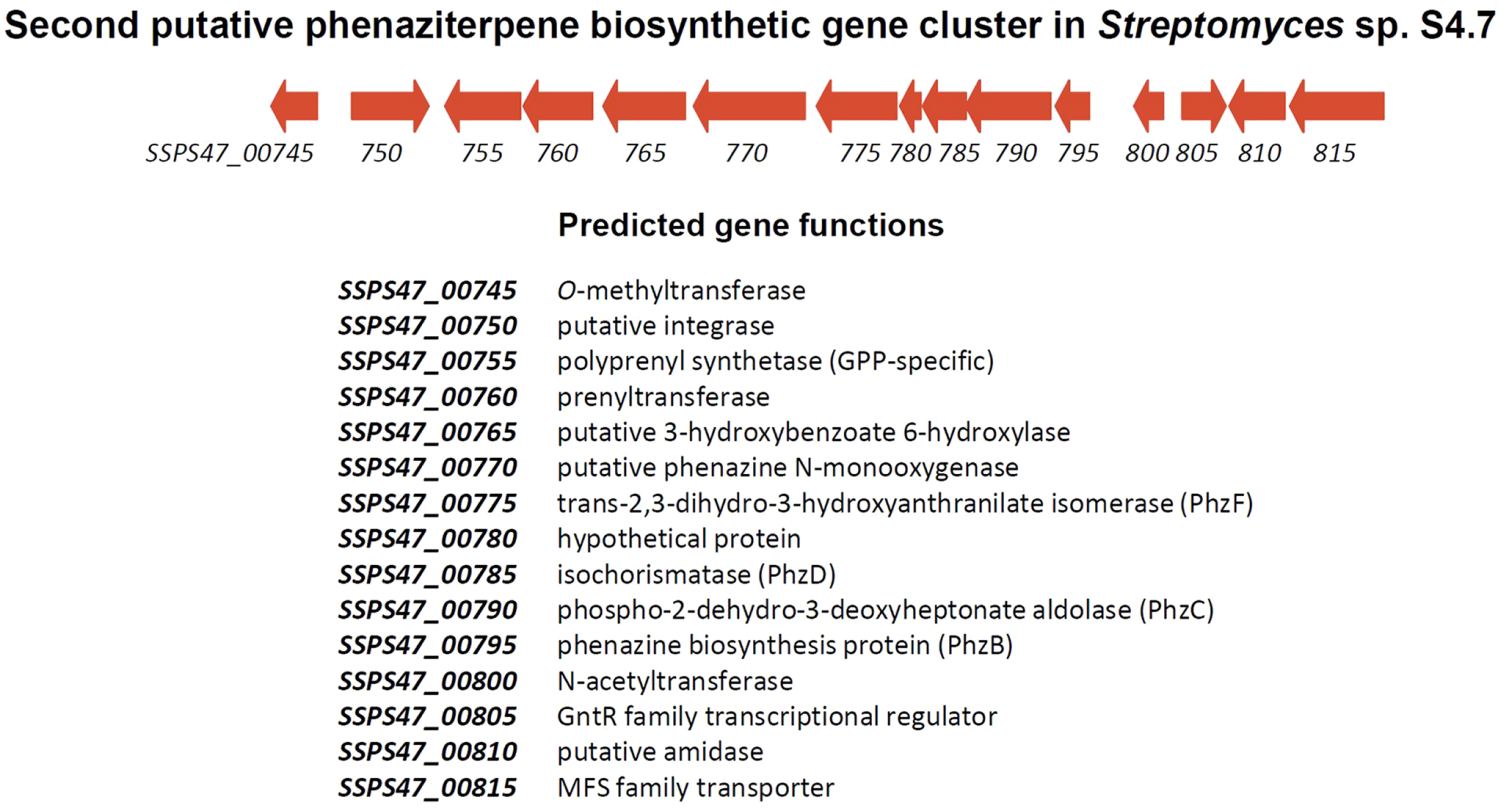
Organization of the second putative phenaziterpene biosynthetic gene cluster in the genome of *Streptomyces* sp. S4.7.

We were unable to detect phenazine production in cultures of *Streptomyces* sp. S4.7, indicating that these clusters, or at least one of them, are silent or nonfunctional under the tested conditions. A analysis of the genome of *S. coelicolor* M1154 used for heterologous expression of the *mfq* cluster revealed no gene encoding a PhzF homologue. To induce heterologous biosynthesis of phenaziterpenes, the *phzF* gene from the second putative phenaziterpene BGC of *Streptomyces* sp. S4.7 (SSPS47_00775) was amplified from the genomic DNA of *Streptomyces* sp. S4.7 and cloned under the control of the strong constitutive PermE* promoter. Extracts from recombinant *S. coelicolor* strains harboring *pCLY10:mfq* and coexpressing *phzF* and *mfqF* were found to contain, in addition to marfuraquinocins and flaviolin, PDC, an apparent intermediate in phenaziterpene biosynthesis (Figures S20 and S21). In contrast, no *mfq*-related metabolites were detected in the strain lacking overexpression of the SARP regulator *mfqF*, strongly suggesting that this regulator is essential for activating both, the meroterpenoid and phenazine pathways (strain 6 and 7 in Table 2). A similar experiment was performed with an *S. coelicolor* strain carrying a modified *mfq* cluster lacking the prenyltransferase-encoding gene *mfqW* (strain 14 in Table 2). This strain showed enhanced PDC production compared to the strain carrying the native, unmodified cluster, suggesting that deletion of *mfqW* may redirect metabolic flux toward PDC synthesis, potentially increasing the yield of phenazine-related compounds. The presence of PDC in the extracts was confirmed by LC-MS through comparison of the standard PDC with the crude extracts (Figure S23, Supporting Information).

### First Insights into the Regulation of the Phenazine Biosynthetic Pathway

While our results clearly demonstrate that both, the meroterpenoid and phenazine biosynthetic pathway of the *mfq* BGC are coactivated by the SARP regulator MfqF, and that coexpression of a *phzF* gene from another BGC is required to biosynthesize the phenazine core structure, we were puzzled by the fact that we could not detect any prenylated phenazines. To rule out the possibility that the *mfqM* gene, presumed to encode a phenazine-specific prenyltransferase, was not being expressed, even after coexpression of *mfqF*, we used the CRISPR/Cas9 system to replace the native promoter region upstream of *mfqM* with the constitutive P*ermE* promoter. The modified cluster was then introduced into an *S. coelicolor* strain coexpressing *phzF* and *mfqF*. However, this manipulation did not lead to the production of phenaziterpenes (data not shown). One possible reason is that *S. coelicolor* M1154 may be unable to supply geranyl pyrophosphate, the presumed substrate required for prenylation of the phenazine core. Alternatively, other enzymes encoded by the second phenazine BGC in *Streptomyces* sp. S4.7 (Figure 5B), might be required for geranylation of the phenazine core, which would involve decarboxylation and subsequent hydroxylation of PDC.

Next, we tested whether MfqQ, a potential regulatory protein whose overexpression and deletion neither had an observable effect on marfuraquinocin biosynthesis, selectively regulates the phenazine biosynthesis. For that, we measured the metabolite profile of an *S. coelicolor* strain with deleted *mfqQ* gene (pCLY10*::mfq:ΔmfqQ*) that was overexpressing both, *mfqF* and *phzF*. This strain produced marfuraquinocins and flaviolin, but not PDC (strain 8 in Table 2). MfqQ contains a SnoaL-like domain, and SnoaL-like proteins are known to function as cyclases in polyketide biosynthesis.^30^ However, when fused to the DNA-binding domain of a σ^70^-like protein, as demonstrated for the sigma factor SigG1 in *Streptomyces tsukubaensis*,^31^ such a protein may act as a metabolite sensor. It seems likely that MfqQ is involved in regulation of the phenazine biosynthesis pathway in response to a certain metabolite, which may be represented by marfuraquinocins precursors, either by repressing its transcription or by being unable to initiate transcription when bound to such ligand. In this way, MfqQ could function as an important regulatory switch between the two distinct biosynthetic pathways encoded by this hybrid biosynthetic gene cluster. However, this hypothesis requires further experimental validation.

In conclusion, a hybrid biosynthetic gene cluster from *Streptomyces* sp. S4.7, specifying a complete biosynthetic pathway for the meroterpenoids marfuraquinocins and a partial one for phenazines, was investigated through heterologous expression, gene inactivation/overexpression, and pathway complementation. A new marfuraquinocin congener with significant antibacterial activity was discovered and characterized. Results from gene inactivation experiments allowed for the proposal of a plausible biosynthetic pathway for marfuraquinocins, providing a foundation for further detailed studies. The phenazine pathway was complemented using a gene from another cluster in the *Streptomyces* sp. S4.7 genome, resulting in activation of phenazine biosynthesis under heterologous expression conditions. We believe that production of the phenaziterpenes reported by Song et al.^16^ may be due to a combined action of enzymes encoded by *mfq* BGC and the other phenazine-specifying BGC (Figure 5B), which also encodes a prenyltransferase. This would explain why we did not detect prenylated phenazines upon heterologous expression of the *mfq* BGC in *S. coelicolor*, even with coexpressed *phzF*.

Interestingly, both the meroterpenoid and phenazine pathways are positively regulated by a single SARP protein, MfqF, while another putative sigma factor-like regulator, MfqQ, appears to be needed specifically for phenazine production. In summary, this study lays the groundwork for further dissection of marfuraquinocin biosynthesis and its potential cross-regulation with phenazine and phenaziterpene biosynthetic pathways.

## Materials and Methods

### Strains, Plasmids, and Media

All strains and plasmids used in this study are listed in Supplementary Tables S1 and S2. *Escherichia coli* XL1-Blue was used as the general cloning host, *Saccharomyces cerevisiae* strain BY4742 was employed for BGC assembly, and *S. cerevisiae* strain TC3 was used for CRISPR/Cas9-mediated gene editing. *E. coli* ET12567/pUZ8002 was used to facilitate the conjugative transfer of nonmethylated DNA to *Streptomyces*, as previously described.^32^

For selection, *E. coli* strains were grown on Luria-Bertani (LB) agar or in liquid LB medium supplemented with appropriate antibiotics. BY4742 was cultured in complete medium (YPD) containing 2% glucose and in yeast synthetic drop-out medium (YSM) Y1376 (Sigma), while strain TC3 was grown in media Y1876 and Y1771. All YSMs were supplemented with 2% glucose.

### Standard Molecular Biology Techniques

Standard molecular biology techniques were performed according to previously published methods.^33^ Plasmid DNA isolation from *E. coli* and DNA restriction/ligation were carried out following the protocols provided by the manufacturers of the kits, enzymes, and reagents (Qiagen, Promega, NEB, and Thermo Fisher Scientific). PCR reactions were conducted using Q5 High-Fidelity DNA Polymerase (NEB). Primers were synthesized by Eurofins and are listed in Table S3 (Supporting Information).

To generate dsOligo fragments for CRISPR/Cas9 reactions, splicing by overlap extension PCR (SOEing) was performed, ensuring at least 20 bp overlapping ends. The standard protocol for Q5 High-Fidelity DNA Polymerase was used for SOEing PCR, and the annealing temperature was predicted using the Clone Manager 10 software.

### Construction and Screening of a Genomic Library

A genomic library of *Streptomyces* sp. S4.7 was constructed according to the manufacturer’s instructions for the CopyControl Fosmid Library Production Kit (Epicenter). Approximately 40 kb DNA fragments were recovered and ligated into the pCC1FOS vector. The ligation product was then packaged using MaxPlax Lambda Packaging Extracts (Epicenter) and transfected into *E. coli* EPI300, generating a genomic library composed of 912 cosmids.

To identify cosmids containing the *mfq* BGC, the library was screened by PCR using three pairs of primers: F1_fwd/F1_rev, F2_fwd/F2_rev, and F3_fwd/F3_rev (Table S3, Supporting Information). Sequencing confirmed that the 31,844 bp *mfq* cluster was split between two cosmids, 13H27 and 8G4.

### Transformation-Associated Recombination (TAR) in Yeast

To construct a specific capture vector for the TAR reaction, flanking regions of 541 bp and 553 bp were synthesized by BioCat, incorporating *Hin*dIII*/NotI* restriction sites for cloning into the pCLY10 vector. A *PmeI* restriction site was also introduced between the flanks to allow for later linearization of the vector for the assembly reaction. The two cosmids carrying fragments of the *mfq* cluster, 13H27 and 8G4, were digested with restriction enzymes as follows: 13H27 was treated with *AclI/PspXI*, while 8G4 was digested with *Pst*I. Fragments of 26,599 bp and 30,571 bp were extracted from the gel for each cosmid, respectively. Three linearized fragments (pCLY10 with flanks digested by *PmeI*, the 26,599 bp fragment, and the 30,571 bp fragment) were cotransformed into *Saccharomyces cerevisiae* BY4742 for the assembly reaction, following the protocol described by Gietz and Woods.^34^ This resulted in the successful construction of the pCLY10:*mfq* plasmid (43,595 bp), which carries the *mfq* cluster.

### Constitutive Expression of *mfqS* and *mfqM* via Promoter Exchange

To express *mfqS*, a phenazine biosynthesis protein (PhzB2), and *mfqM*, a putative aromatic prenyltransferase, the native promoters in actinobacteria were replaced with the constitutively expressed *ermE* promoter.

To achieve this, a dsOligo was generated using SOEing PCR for the *mfqSermEup* construct. Three fragments were amplified: *mfqS1* and *mfqS2* from the native *mfq* cluster, and the *pEM* fragment, which contains the *ermE* promoter, from the pUWLoriT vector (Table S4, Supporting Information). The following primers were used: for the *mfqS1* fragment (356 bp): mfqS1_pEm_fwd/mfqS1_pEm_rev; for the *mfqS2* fragment (418 bp): mfqS2_pEm_fwd/mfqS2_pEm_rev; for the *ermE_up* fragment (426 bp): pEm_fwd/pEm_rev.

All three fragments were fused by SOEing PCR, resulting in a 1,157 bp *mfqSermEup* fragment with homologous flanks for insertion into the pCLY10:*mfq*, replacing the native *mfqS* promoter.

The dsOligo *mfqMermEp* (1,222 bp) was synthesized by BioCat and consisted of two flanks derived from the MTP/PPH cluster (430 bp and 482 bp) and the *ermEp* promoter fragment (310 bp). The dsOligo was introduced into the MTP-PPH vector via TAL assembly. Before the reaction, the vector was linearized with *NdeI* for the insertion of *mfqMermEp* or with *PspXI* for promoter replacement in the *mfqS* gene.

### Overexpression of Putative Regulatory Genes

*In silico* analysis revealed three putative regulator genes within the MTP/PPH cluster that may be responsible for the production of marfuraquinocin and/or phenazine compounds. In this study, the *mfqF, mfqQ*, and *mfqL* genes were overexpressed using the pUWLoriT vector. The genes were amplified from genomic DNA using the following primer sets: SARP_*Bam*HI/SARP_*Hin*dIII for *mfqF;* mfqL_*Bam*HI/mfqL_*Hin*dIII for *mfqL;* mfqQ_*Eco*RI/mfqQ_*Hin*dIII for *mfqQ.*

The *mfqF* and *mfqL* genes were cloned into the pUWLoriT vector using *Bam*HI*/HindIII* restriction sites, while the *mfqQ* gene was cloned using *Eco*RI*/HindIII*. The resulting vectors (pOE_SARP, pOE_mfqL, and pOE_mfqQ) were individually coexpressed with the pCLY10:*mfq* vector. The *phzF* gene, which was suggested to be essential for phenazine core production, was amplified using the phzF_*Hin*dIII/phzF_XbaI primer set and cloned into the pUWLoriT vector, generating pOE_phzF.

To allow constitutive expression of *phzF* alongside *mfqF* and *mfqL*, the ermE-mfqF and ermE-mfqL fragments from pOE_SARP and pOE_mfqL were amplified using the pOE_mfqL_XbaI/pOE_SARP_SacI and pOE_mfqL_XbaI/pOE_mfqL_SacI primer sets, yielding fragments of 1,705 bp and 649 bp, respectively. After ligation at the *XbaI/SacI* restriction sites, these fragments were inserted downstream of the *phzF* gene, generating the vectors pOE_phzF-mfqL and pOE_phzF-SARP, in which expression is independently driven by two PermE* promoters.

### Gene Inactivation with CRISPR/Cas9 Technology

CRISPR/Cas9 technology was used to deactivate four genes (*mfqQ, mfqH, mfqM,* and *mfqW*) in the pCLY10:*mfq* vector, following the method described by Jakočiu̅nas et al.^22^ The freely available CRISPy tool (http://staff.biosustain.dtu.dk/laeb/crispy_cenpk/) was used to identify the PAM sequences and select specific sgRNAs.

CRISPR/Cas9 gene editing was performed in *Saccharomyces cerevisiae* TC-3, which expresses the Cas9 protein. The sgRNAs were expressed using the p426 vector and were introduced via PCR amplification using the following forward primers: p426_mfqH263-P, p426_mfqM236-P, p426_mfqQ471-P and p426_mfqW401-P. A common reverse primer, *p426_rev-P*, was used for all reactions. All primers, synthesized by Eurofins, had phosphorylated 5′ ends, allowing for direct blunt-end ligation of the amplified p426 vector using T4 DNA Ligase (NEB). The accuracy of the constructs was confirmed by sequencing with the p426_seq primer.

### Heterologous Production and Purification of Meroterpenoids

pCLY10:*mfq* and modified versions thereof were introduced into *Streptomyces coelicolor* M1154 and *Streptomyces albus* DEL14 strains via conjugation. The production of meroterpenoids and phenazines was tested in different media, including: MYM (maltose 4 g/L, yeast extract 4 g/L, malt extract 10 g/L, tap water 0.5 L, ddH_2_O 0.5 L, adjusted to pH 7.3, autoclaved, and supplemented with 2 mL/L trace elements according to the R2YE preparation protocol,^35^ SM17 (Ye et al. 2017),^100^ and MP1 (glucose 40 g/L, yeast extract 1.5 g/L, NH_4_NO_3_ 2.5 g/L, MgSO_4_·7H_2_O 0.5 g/L, KH_2_PO_4_ 0.5 g/L, CaCO_3_ 3 g/L) and liquid SFM medium (soya flour 20 g/L, D-mannitol 20 g/L).

Recombinant strains M1154/*mfq*/pOE_SARP and DEL14/*mfq*/pOE_SARP were cultured in 2xYT and TSB media,^35^ supplemented with 30 μg/mL thiostrepton and 15 μg/mL apramycin, respectively. In 250 mL baffled flasks, 50 mL of medium was inoculated with 50 μL of spore suspension and cultured at 200 rpm for 3 days. This was followed by inoculation of 50 mL of production medium with a 5% inoculum and cultivation for an additional 10 days. Optimal meroterpenoid production was observed in MYM medium inoculated with the M1154/*mfq*/pOE_SARP strain.

To scale up meroterpenoid production, 60 × 50 mL of MYM medium in 250 mL baffled flasks was cultivated as described above. The pellet was separated by filtration using 240 mm filter paper (Macherey-Nagel GmbH), washed twice with ddH_2_O, and extracted with 80% acetone for 24 h at 4 °C. The organic fraction was separated by centrifugation and concentrated using a rotary evaporator.

The extract from the pellet was subjected to semipreparative HPLC using a Shimadzu system consisting of a CBM-20A system controller, two LC-20AR solvent delivery pumps, a DGU-20A3R degasser, a manual injector, an SPD-10Ai UV–vis detector, and an FRC-10A fraction collector. Fractionation was performed using a Shim-pack GIS column (Shimadzu, Nakagyo-ku, Kyoto, Japan), 5 μm, 20 × 250 mm specifications. A gradient elution was applied using solution A: ddH_2_O; solution B: ACN (LiChrosolv Reag. Ph Eur, gradient grade for LC, Merck KGaA).

Elution was carried out as follows: 0–10 min: 50% B; 10–20 min: 95% B; room temperature with a flow rate of 20 mL/min and an injection volume of 1 mL. The detection wavelength for fractionation was set at 254 nm. From 1.2 g of crude acetone extract, 6.5 mg of compounds were purified, with an elution time of 15 min. MS analysis confirmed the presence of at least two compounds, one of which was a meroterpenoid.

For further purification, semipreparative HPLC with an optimized gradient (0–45 min from 5% B to 95% B, followed by a washing step at 95% B for 10 min) was used. The peak at 32 min was collected, yielding 3.2 mg of compound **1**.

### Secondary Metabolite Identification and Characterization

LC-MS analyses were performed using either of two instruments: a Vanquish Horizon UHPLC system coupled to the ESI source of an LTQ Orbitrap Velos mass spectrometer (both from Thermo Fisher Scientific) or another Vanquish Horizon UHPLC system coupled to the ESI source of a timsTOF fleX mass spectrometer (Bruker Daltonics). Chromatographic and MS parameters for the Orbitrap instrument were as previously described.^36^

For the Qq-TOF instrument, separation was carried out using an Acquity Premier HSS T3 column (2.1 × 150 mm, 1.8 μm, Waters). The mobile phases consisted of solution A: water with 0.1% formic acid; solution B: acetonitrile/water (9:1) with 0.1% formic acid.

The sample components were separated and eluted using the following gradient: 0–10 min: linear increase from 0% to 20% B; 10–25 min: linear increase from 20% to 100% B; 25–28 min: isocratic column cleaning at 100% B; 28–33 min: re-equilibration at 0% B.

The flow rate was set to 0.5 mL/min, and the column oven temperature was maintained at 40 °C. High-resolution ESI-MS and MS/MS spectra were recorded in positive ion mode over an *m*/*z* range of 100–2500. CID spectra of the five most intense precursor ions in each MS1 spectrum were acquired in automated data-dependent acquisition mode using nitrogen as the collision gas. The sum formulas of the detected ions were determined using Bruker Compass DataAnalysis 5.3, based on mass accuracy (Δ*m*/*z* ≤ 5 ppm) and isotopic pattern matching (SmartFormula algorithm).

^1^H and ^13^C (DEPTq) 1D, as well as COSY, HSQC, HMBC, and NOESY 2D NMR spectra of marfuraquinocin E (3.2 mg) in CDCl_3_ at 298 K, were recorded on an Avance III 600 NMR spectrometer (Bruker BioSpin) equipped with an N_2_ cryo probe (Prodigy BBFO with z-gradient; 600.25 MHz for ^1^H, 150.93 MHz for ^13^C). Chemical shifts were calibrated using the residual ^1^H solvent signal at δ = 7.24 and the ^13^C solvent signal at δ = 77.23.

### Mammalian Cell Culture

HepG2 cells were purchased from the American Type Culture Collection (ATCC, Manassas, VA, USA) and were cultured in DMEM (Lonza) supplemented with 2 mM l-glutamine (Merck) and 10% heat-inactivated fetal bovine serum (FBS) obtained from Biowest. Human colon carcinoma HCT116 cells were from the American Type Culture Collection (ATCC, Manassas, VA, USA) and cultured in Mc Coy’s 5A with l-glutamine (Merck) supplemented with 10% FBS. Both cell lines were passaged every 2–3 days and used until passage number 30. HUVECs (pooled donors) were purchased from Lonza and were maintained in M199 (Lonza) supplemented with 2 mM l-glutamine (Merck), 20% FBS and 0,4% ECGS + 90 μg/mL heparin (PromoCell). Cells were kept in precoated (1% gelatin in phosphate-buffered saline) cell culture flasks, passaged at a ratio of 1:3 and used between passage number 2 and 5. To all media 100 U/mL penicillin + 100 μg/mL streptomycin (Lonza) was added and cells were cultured at 37 °C and 5% CO_2_. Viability and cell number was monitored using an automated cell counter (Vi-CELLXR Cell Viability Analyzer, Beckmann Coulter GmbH).

### Bioactivity Tests

The antimicrobial activities of the extracts were tested against *Escherichia coli* DH5αF’, *Pseudomonas putida* KT2440, *Micrococcus luteus* DSMZ 1790, *Bacillus subtilis* DSMZ 10, and *Saccharomyces cerevisiae* BY4742. All bacterial strains were cultured in liquid LB medium for stock solution preparation (20% glycerol, v/v) and on LB-agar plates for bioassay experiments. *E. coli* plates were incubated at 37 °C for 18 h, while the other bacterial strains were incubated at 30 °C for 18 h. *S. cerevisiae* BY4742 was grown in YPD liquid medium and on YPD agar plates at 30 °C.

Sterile filter discs (9 mm in diameter, Whatman filter paper) were impregnated with a dimethyl sulfoxide (DMSO) solution containing 1 μg of compound **1**. The impregnated discs were dried for 15 min under sterile conditions and then placed on the agar surface seeded with microbial culture. After 18 h of incubation, the inhibition zone around each disc was measured in millimeters (mm).

The minimum inhibitory concentration (MIC) for marfuraquinocin E against *Micrococcus luteus* was determined using a broth microdilution assay in a 96-well microtiter plate format. An overnight culture of *M. luteus* was prepared by incubating the strain in 5 mL of TSB at 28 °C with agitation. The following day, the culture was diluted with fresh TSB to an optical density at 600 nm of 0.06. A volume of 200 μL of the diluted culture was dispensed into each well of a sterile, flat-bottom 96-well plate. The marfuraquinocin E, prepared as a stock solution in DMSO, was added to achieve final concentrations ranging from 0 to 60 μg/mL. Wells containing DMSO only served as solvent controls. Each concentration was tested in triplicate. Plates were incubated at 28 °C for 18 hours, after which bacterial growth was assessed by measuring OD_600_ using a Multiskan GO microplate spectrophotometer (Thermo Fisher Scientific). The MIC was defined as the lowest concentration of marfuraquinocin E that completely inhibited bacterial growth.

Cell viability and proliferation/cytotoxicity were assessed using a resazurin conversion assay^37^ followed by crystal violet staining in HepG2 cells, HCT116 cells, and primary HUVECs. For the resazurin conversion assays, cells were treated with DMSO (Carl-Roth) serving as solvent control and marfuraquinocin E at the indicated concentrations. Paclitaxel was obtained from Sigma-Aldrich and was used as positive control at a concentration of 3 μM. After 48 h of compound treatment, cells were incubated for 1 h in serum-free medium containing 10 μg/mL resazurin (Sigma-Aldrich). Resazurin conversion was measured in a 96-well plate reader (Tecan GENios Pro) by monitoring fluorescence at 590 nm (excitation wavelength: 535 nm). Afterward the crystal violet-stained biomass was quantified by spectrophotometry at 595 nm using a TECAN Sunrise reader.

### Heterologous Expression mfqw in *E. coli*

To test the in vitro prenyltransferase activity of MfqW, this protein was expressed in *Escherichia coli* BL21­(DE3) (Invitrogen, Carlsbad, CA, USA) carrying the pET-MfqW vector. To ensure efficient expression in *E. coli*, the *mfqW* gene was codon-optimized, synthesized by BioCat GmbH (Heidelberg, Germany), and fused with an N-terminal 6 × His tag. The gene *mfqW*-His was cloned into the pET30a+ expression vector using the restriction sites *NdeI*/*BamH*I.

Protein expression and isolation were performed as described by Schneider et al.,^38^ with minor modifications. Briefly, an overnight culture grown at 37 °C in LB medium containing kanamycin was used to inoculate 200 mL of LB medium supplemented with the same antibiotic. The culture was incubated at 37 °C until it reached an optical density at 600 nm of approximately 1.0, after which overexpression was induced with 0.5 mM isopropyl-β-d-thiogalactopyranoside (IPTG). Following induction, the culture was incubated for 2 h at 25 °C and then overnight at 15 °C with shaking at 220 rpm. Cells were harvested by centrifugation (15 min, 4,000*g*, 4 °C). The cell pellet was resuspended in 10 mL of lysis buffer (50 mM potassium phosphate buffer, pH 8.0; 300 mM NaCl; 10 mM imidazole; 1 mM dithiothreitol [DTT]; 100 μg/mL lysozyme) and disrupted using a Bioruptor Plus sonicator (amplitude 30%, cycle 3, 10 min). The lysate was clarified by centrifugation (30 min, 10,000 rpm, 4 °C), and the supernatant was filtered through a 2 μm membrane to remove cell debris. The recombinant His-tagged MfqW protein was purified using Ni-nitrilotriacetic acid (NTA) affinity chromatography (Qiagen) according to the manufacturer’s instructions, with the following buffer modifications: washing buffer (50 mM potassium phosphate, pH 8.0; 300 mM NaCl; 20 mM imidazole; 1 mM DTT), elution buffer (50 mM potassium phosphate, pH 8.0), and dialysis buffer (50 mM Tris-HCl, pH 8.0; 50 mM NaCl). Protein purity and concentration were analyzed by SDS-PAGE and the Bradford assay, respectively.

### In Vitro Assay and LC–MS Analysis of mfqW Enzyme Activity

The in vitro assay for MfqW enzyme activity was adapted from Noguchi et al.^23^ with minor modifications. Enzymatic reactions were carried out at 30 °C for 1 h in a total volume of 200 μL containing 50 mM HEPES–NaOH buffer (pH 7.5), 5 mM MgCl_2_, 200 μM 2-O-PHN, 200 μM of the respective prenyldiphosphate substrate (DMAPP, GPP, or FPP), 5 mM sodium dithionite, and 2 μM purified MfqW. After incubation, the mixtures were centrifuged to remove precipitates, and the resulting supernatants were subjected to LC–MS analysis.

Chromatographic separation was performed on an LC system using solvent A (H_2_O + 0.1% formic acid) and solvent B (acetonitrile + 0.1% formic acid) at a flow rate of 0.45 mL min^–1^. The gradient was programmed as follows: 95% A for 0–0.5 min, linearly decreasing to 0% A over 18 min, held for 2 min, and re-equilibrated to initial conditions for 2.5 min.

Mass spectrometric detection was conducted in positive ion mode (*m*/*z* 100–1000 Da). Ion source parameters were as follows: gas 1, 55 psi; gas 2, 50 psi; curtain gas, 35 psi; source temperature, 500 °C; declustering potential, 80 V. IDA mode was used for small-molecule analysis, selecting up to five precursor ions (intensity threshold 5 cps) with dynamic background subtraction and 2 s dynamic exclusion. MS^2^ spectra were acquired using a collision energy of 35 ± 15 V.

### Identification of Phenazine 1,6-Dicarboxylic Acid (PDC) in Crude Extracts by LC–MS/MS

LC–MS/MS analysis of PDC was performed under conditions similar to those described for analysis of MfqW enzyme activity, with the following modifications: the mass range was set to *m*/*z* 100–1600, the ion source temperature was 520 °C, ion source gas 1 was 50 psi, gas 2 was 70 psi, and the curtain gas was 30 psi. Crude extracts were compared with a PDC standard (10 μM; Sigma-Aldrich) based on retention time and MS/MS fragmentation patterns.

## Supplementary Material



## Notes

Tables S1–S4: Bacterial strains, plasmids, oligonucleotides and DNA fragments used in this study; Method S1: description of 2-*O*-methylflaviolin synthesis; Figure S1: Confirmation of mfqBGC assembly in pCLY10 vector; Figures S2–S4, S16–S21: Base peak chromatograms and extracted ion chromatograms of the LC-MS analysis of culture extracts from various strains; Figure S5: High-resolution ESI-Qq-TOF MS and MS/MS spectra of marfuraquinocin E; Figures S6–S12: ^1^H, ^13^C (DEPTq), COSY, HSQC, HMBC, and NOESY NMR spectra of marfuraquinocin E; Figure S13: Assay demonstrating antibiotic activity of marfuraquinocin E; Figure S14: Determination of MIC of marfuraquinocin E; Figure S15: Assessment of marfuraquinocin E cytotoxicity; Figure S22: In vitro prenylation assay of mfqW; Figure S23: Identification of the phenazine-1,6-dicarboxylic acid (PDF)

## #

Institute of Science and Technology Austria (ISTA), Am Campus 1, 3400 Klosterneuburg, Austria
