# Supplementary material for: Heterologous Expression and CRISPR/Cas9-Assisted Manipulation of the Hybrid Gene Cluster Specifying the Biosynthesis of Meroterpenoids and Phenazines
Source: ACS Synth Biol. 2025 Dec 23;15(1):137–48. doi: 10.1021/acssynbio.5c00531 (PMC12814520; doi:10.1021/acssynbio.5c00531)
Supplement: Supplementary file 1 [file sb5c00531_si_001.pdf]

Supporting Information

Heterologous expression and CRISPR/Cas9-assisted manipulation of the hybrid gene cluster specifying biosynthesis of meroterpenoids and phenazines

Olha Schneider[a], Martin Zehl[b,f], Margherita Miele[c], Vittorio Pace[d], Corinna Brungs[a], Jan-Fang Cheng[e], Scarlet Hummelbrunner[a], Verena M. Dirsch[a], Sergey B. Zotchev[a]*

[a] Department of Pharmaceutical Sciences, Division of Pharmacognosy, University of Vienna, 1090 Vienna, Austria

[b] Department of Analytical Chemistry, Faculty of Chemistry, University of Vienna, 1090 Vienna, Austria

[c]Department of Chemistry, University of Turin , Via P. Giuria 7, 10125 Turin, Italy

[d]Department of Chemistry, University of Rome "La Sapienza", P.le A. Moro, 5 - 00185 Rome, Italy

[e]US Department of Energy Joint Genome Institute, Lawrence Berkeley National Laboratory, Berkeley, CA 94720, USA

[f] Current address: Institute of Science and Technology Austria (ISTA), Am Campus 1, 3400 Klosterneuburg, Austria

Table S1. Bacterial strains used in this work.

Bacterial strain	Description	Source
E. coli XL-1 Blue	**General cloning host:** *endA1*, *hsdR17* (rk⁻, mk⁺), *supE44*, *thi* ⁻, λ⁻, *recA1*, *gyrA96*, *relA1*, (*lac*), [F', *proAB*, *lacI^q*, *lacA*ΔM15, *Tn10^Tc*]	Agilent Technologies
Escherichia coli EPI300	**Subcloning host:** *F* ⁻*, mcrA, Δ(mrr-hsdRMS-mcrBC), Φ80dlacZΔM15, ΔlacX74, recA1, endA1, araD139, Δ(ara leu)7697, galU, galK, λ* ⁻*, rpsL* (Str^R), *nupG, trfA, dhfr*	Lucigen Corporation
E. coli ET12567/pUZ8002	Strain for intergenic conjugation; RP4 oriT with helper plasmid pUZ8002 (Kan^R, Cm^R); methylation deficient (*dam*−, *dcm*−, *hsdM*−)	1
Saccharomyces cerevisiae BY4742	derivative of S288C, (*Mat α*; *his3*Δ1; *leu2*Δ0; *lys2*Δ0; *ura3*Δ0)	2
Saccharomyces cerevisiae TC-3	MATa *his3*Δ1 *leu2*Δ0 *met15*Δ0 *ura3*Δ0 *trp1*Δ0, P414 - TRP1-TEF1p-Cas9-CYC1t	3

Streptomyces coelocolor M1154	Derivate of *Streptomyces coelicolor* M145 [wild-type strain A3(2) lacking plasmids SCP1 and SCP2), Δ*act*; Δ*red*; Δ*cpk*; Δ*cda*; *rpoB*(C1298T); *rpsL*(A262G)	4
Streptomyces albus DEL14	Derivative of *Streptomyces albus* J1074 with deletion of 15 gene clusters	5

Table S2. Plasmids used in this work.

Plasmid	Description	Source
pUWLoriT	Replicatable shuttle plasmid pIJ101 minimal replicon, Thio^R, Amp^R, RP4 *oriT*, *ColEI* replication origin, *ermE**p	6
pCLY10	*oriT, attP, VWBint, ori15A,* Amp^R*, CEN6-ARS4, LEU2*	7
p426	*Ura3-SNR52p-gRNA.CAN1.Y-SUP4t*	#43803, Addgene
pOE_*mfqF*	pUWLoriT expressing *mfqF* gene, encoding for SARP regulator; cloning over *BamH*I/*Hind*III	This work
pOE_*mfqL*	pUWLoriT expressing *mfqL* gene, encoding for a putative helix-turn-helix domain protein; cloning over *BamH*I/*Hind*III	This work
pOE_*phzF*	pUWLoriT expressing *phzF* gene, encoding for an essential enzyme of phenazine biosynthesis, catalysing the isomerization reaction, originating from cluster1; cloning over *Hind*III/*Xba*I	This work
pOE_*mfqL-phzF*	pUWLoriT expressing *mfqL* and *phzF* genes	This work
pOE_*mfqF-phzF*	pUWLoriT expressing *mfqF* and *phzF* genes	This work
pOE_*mfqQ*	pUWLoriT expressing *mfqQ* gene, encoding for a putative sigma-70 family protein; cloning over *Hind*III/*EcoR*I	This work
pCLY10::mfq	pCLY10 vector carrying MTP/PPH biosynthetic cluster	This work
*pCLY10::mfq*Δ*mfqQ*	pCLY10 vector carrying MTP/PPH biosynthetic cluster, with deleted *mfqQ*	This work
*pCLY10::mfq*Δ*mfqH*	pCLY10 vector carrying MTP/PPH biosynthetic cluster, with deleted *mfqH*	This work
*pCLY10::mfq*Δ*mfqM*	pCLY10 vector carrying MTP/PPH biosynthetic cluster, with deleted *mfqM*	This work
*pCLY10::mfq*Δ*mfqW*	pCLY10 vector carrying MTP/PPH biosynthetic cluster, with deleted *mfqW*	This work
pET-MfqW	pET30a(+) vector carrying *mfqW* gene, cloned via *Nde*I/*BamH*I restriction sites.	This work

Table S3. Oligonucleotides used in this study. Italic indicates an endonuclease restriction enzyme site, underlined is the sequence complemental to the p426 vector.

Primer name	Primer sequence
F1_fwd	GCCAACTCGCCGCGTAG
F1_rev	GCAGTTTGGTGGTCGAAACG
F2_fwd	CCGCTGATCGGTGAATTCG
F2_rev	ATGGAGTACTGCGGCCAGAG
F3_fwd	GCGTGTCGACCATGGGATAC
F3_rev	GCTTGACCTCGCGTACGTAG
BamHI_SARP	*GAAGGATCC* GACCTGTGGCCTGTGATTC
HindIII_SARP	*GCCAAGCTT* TCCCACAGCGTGTACATCCC
mfqQ_EcoRI	*GAGCGAATTC* ACGCCAGTTTTCGCAACATG
mfqQ_HindIII	GCGGTCAAGCTTGTGACGTG
mfqL_HindIII	*GGCAAGCTTCTCAATACCCGGGCGAAGG*
mfqL_BamHI	CTGCTGAAGGAGCGTCAAG
phzF_HindIII	GTACAAGCTTCTCACCGACACGACCGATTGG
phzF_XbaI	GATCTCTAGACTGCTCAGGTGTCTGCCGTC
pOE_mfqL_XbaI	TCGTTCTAGACCGCGGGAGTAATCCTGGG
pOE_mfqL_SacI	TAATGAGCTCTCGAGCGATCCGGGAGAGG
pOE_SARP_SacI	TCGTTGAGCTCGACCTGTGGCCTGTGATTCC
*mfqQ1*_fwd	CGCTATCGCAACCACTTCATC
*mfqQ1*_rev	GTGATGCGTCATGAAGCCTCC
*mfqQ2*_fwd	GGAGGCTTCATGACGCATCACGCCAATCCCGACAAACTCTCT
*mfqQ2*_rev	GTCCTCGGACTTCAAGGGACC
*mfqQ*_seq_fwd	GGAGACCAGTCAGCAGATCAC
*mfqQ*_seq_rev	TGAAGCTGGTCTGCGTGTTG
*mfqH1*_fwd	CATTCCGAACACCGAGGACTG
*mfqH1*_rev	GATGAGCCGCATGATCCGATC
*mfqH2*_fwd	GATCGGATCATGCGGCTCATCACGTTGGTCTTCGCCCGTTAG
*mfqH2*_rev	CAAGGTGTACTCGGTCTTCAC
*mfqH*_seq_fwd	GCAACGTCCTGATCGTCTC
*mfqH*_seq_rev	ATACAGGACACCGCCGATG
*mfqM1*_fwd	TTCCTTCGCCCGGGTATTGAG
*mfqM1*_rev	GTGGCTACGGGCATTCAAG

*mfqM2*_fwd	CTTGAATGCCCGTAGCCACTACAAGATCGCGGCCTACCAC
*mfqM2*_rev	TATGAGCACGGGTGTCATCGC
*mfqM*_seq_fwd	CCTTGCCCTGATCCAGATG
*mfqM*_seq_rev	GTGGCTGAGGAGGTTCATC
mfqW1_fwd	CTGATCACGCTGTGCTACG
mfqW2_rev	GGCTCCGGACATTGAATCC
mfqW2_fwd	GGATTCAATGTCCGGAGCCGGCTCGTACTACCAGATCAG
mfqW1_rev	GAACGGGTCGGCTACTACAAC
*mfqW*_seq_fwd	AGCGCTCGGTGTACGAAAC
*mfqW*_seq_rev	ACGGGTACGACCTCGTCATC
*mfqS1*_pEm_fwd	CTCGGCGGGTCTGGTGCTGGC
*mfqS1*_pEm_rev	CAGGATTACTCCCGCGGCTTCGGGCCACACACGGACGGTGG
*mfqS2*_pEm_fwd	CCACTAGTTCTAGAGCGGCCCAGTGGAAAGTGAGGCGGCTC
*mfqS2*_pEm_rev	TGCTGCTTGATCTTGCCGTTC
pEm_fwd	AAGCCGCGGGAGTAATCCTG
pEm_rev	GGCCGCTCTAGAACTAGTGG
pSH_*mfqS*_seq1	CGGCATCGACTACAACATC
pSH_*mfqS*_seq2	TGCGTGGTCGAGGATCTAC
pSH_*mfqM*_seq1	CTGCTGAAGGAGCGTCAAG
pSH_*mfqM*_seq2	ACGTTCTGTGGGCGTAGTC
p426_*mfqH263-P*	CAAAGTCAAGATGGGACACAGTTTTAGAGCTAGAAATAGCA
p426_*mfqM236-P*	GAACGGACTCGTCGAGAAGAGTTTTAGAGCTAGAAATAGCA
p426_*mfqQ471-P*	TTCGTCCTGCACGAGGTGTTGTTTTAGAGCTAGAAATAGCA
p426_*mfqW4*01-P	CGCCGTACCGTCCATGCCGCGTTTTAGAGCTAGAAATAGCA
p426_rev-P	GATCATTTATCTTTCACTGCGGAGAAG

Table S4. DNA fragments used in this study for deletion of *mfqM*, *mfqH*, *mfqQ*, *mfqW*, and replacement of native promoters for *mfqS* and *mfqM* with P*ermE**.

Name	DNA sequence
**Δ*mfqM*_dsOligo**	TTCCTTCGCCCGGGTATTGAGGTGTGCTCAAAATTCCCCCTTCGCGCGTGGATCCACCCACAACCCCCCGGATGCGAAACGCTGGTGGAC<br>CCGCCTGAGGGAGAGGTTCACCGAGACGGGCGGCACCCGTCAAGGGTCCCGGCGAGGGGGTGAACCGGCCCGTCCAACCCCCTTCTCCAC<br>AACCGGAACAAAAGCACGTTCGCATGGATCAAGGTCCCTCACTCTGCCGCTGTCGCCGGTTTCGCGGCAACGACGAGTTCAACGAGCAGG<br>ACGGGTTCGGAGGGGGGCGCGGGTCCGGTGGGGCGACGGGGTCAATCGGAGGGACTTGGCGTGGACCGGTGAGCGAGTTGGTTTCCGCTT<br>GGTGTCGAGCCGGGCCCGAGCCGCCCCCGAGCCGACACCGACCCGCGCGTCGGCGCCAAGCCGACACCAAGCGGGGGACACGACGATCTC<br>GCCATATGCCCGAGCCGGCCCTGGGGGCACAAGCCGGTGGTCCGCATTCCCTTGGAGGCTTGAATGCCCGTAGCCACTACAAGATCGCGG<br>CCTACCACCAGAAGACGGCGCAACTGTCCGACCGGGTGCGCCCCCCGACCCCGAAGTGACGCACGGCCGCACCTCGACAAGCACGCGACA<br>CACCTCGACCAGCAGGCGACACACCTCGACGAACAGGCACCACACCGAGACGGACCACATGAGCAGAAGGGGCAGAACAGCATGACCGGA<br>GTCCATGAACTCGCGCGCCGCCGGGCGGAAGGCCCCGGGACCGGGGAAGCGGAGGAGGAGAAGCGATGACACCCGTGCTCATA
**Δ*mfqH*_dsOligo**	CATTCCGAACACCGAGGACTGGATCTCGTACGCCGTGCGCGACACCGGCTTCCACTTCCAGCTCGACCGCCGGGTACCCGGAACGATGGA<br>ACCGCTCGCGCCGGTGCTGCGCGATTTCGCCGGGAGCCACGGCTGGGACGTCGGCAACCTCGACTTCTACATCATCCACGCCGGCGGTCC<br>GCGGATCCTGGACGATCTGGCCAAGTTCCTCAATGTCGACCGCAAGGTGTTCCGTCACAGCTGGTCCACCCTGGCCGATTACGGAAACAT<br>TGCCAGCGCTGTCGTTCTTGAAGCGCTGCGCAGGCAATTCGAGGAGGACACGATACTGCCGTCGGCCGCCACGGGGATGATCGCCGGCTT<br>CGGTCCCGGCATTACCGCCGAGATGGCACTGGGCCGTTGGACCGTGGAAACGCAAGGAAATCCGGCCGACGCGTCGGCGCCGTATGTGTA<br>TTCGGGGGGGACAATATGAACAGCGGTCGACCGACATCGGATCGGATCATGCGGCTCATCACGTTGGTCTTCGCCCGTTAGCGGGTGTCT<br>CGCGGCGCCCCCGCGGGTCAGAGGGTGCGGTGGCCGGCCTTGAGGGCCGCCGCCACGACGACGGCGCGCCGGGCCTGGTAGTGGGCGGAG<br>TTCAGGACCGCTTCGCCGGGCGGGCCGTCGGCCGCCCAGTGGGAGGTGCCGTACGGATTGCCGACCCCGTTCCCGGCGGGGTCGGTGTAA<br>CCGGGGGGCACGATGACGCCGCCCCAGTGGTAGAACACGTTCCCCAGCGCCAGGATGGTCGACTCGTGTCCGCCGTGCGCGGAGCCGGTG<br>GAGGTGAAGACCGAGTACACCTTG
**Δ*mfqQ*_dsOligo**	CGCTATCGCAACCACTTCATCCACGGCTTCACGCTGAGTGACGGGATGATCGCCGCCAGCCGCGAGTACACCAATCCCATCGAGCACATG<br>CGTGCCCTCAGCATCGAGACACCGCACATCAAGCGTGACTGGATCCCGTCCTGACCTGCCCTTTGGCCCACCGGTCCGGTCGCGGCGGTC<br>AAGCTTGTGACGTGGATCACATTTTTGGAATGGATGTCACACTCCGGCCGCCCGGACAGCTCTCAGGTCCGAACGCAACCGCGACGGGAG<br>GCTTCATGACGCATCACGCCAATCCCGACAAACTCTCTTACGTCGACTGACAACCGAGGAACTCACATGTTGCGAAAACTGGCGTCCCTA<br>CCCAGTGGCAGGAGGGCGAAATGGGTTGTCCTCGCCCTCTGGGTCGTCTTGCTGATTCCCGCCGTCCTGCTGGCCGGGAAGCTCGGCGAC<br>GTGCAGGAGAACGACAACTCGGCCTGGCTGCCGGGTGACGCGGAGTCGACCGCCGTCGTCGAGCGGGCCGAGAAGTTCCAGCCCACCGAC<br>ACCGTGCTGGCGCTCGTCATCTACGACCGGCAGGACGGCGTCACCGCCGCCGACATGGCCAAGGCCCAGGCCGACGTCGAAGCCTTCCAG<br>GGCGTCGAGAAGGTGGTCGGCGAGCCCCAGGGTCCCTTGAAGTCCGAGGAC
**Δ*mfqW*_dsOligo**	GAACGGGTCGGCTACTACAACACCTTCGCCGCCGTCAGCCGCACCGGCCACCGCGATGTGCCGGGGGTGGGACCGGTACGGGTCAGCGGG<br>GACCCGGACACCGGAGAGGTGGACATGCTCCGCGGTCCGCACTTCGCCTCCTTCCAGTTCCACGCCGAGTCGGTGCTGACCGTCGACGGG

	CCCCGGCTGTTCGCCGACGCGCTGCGGGGCGTGCTCGACAGCTGAGGCACAGGACGCAGGGGACGCGCACCCTCGCTCCCGCGAGTGGTG<br>CGCGTCCCCTGCGTCCTGTGCCCGGCGCCGCTCTAGTCCTCCAGAGCGTCGAACGCCTTCACCAACTTCCGCTGGTGATCGCTGATCTGG<br>TAGTACGAGCCGGCTCCGGACATTGAATCCCCCACATGTCGGTCTTGGATGTCTGGTTCGATCGGGCCGGTCGGCCGCCGGTGCACGAGG<br>AACATCGCCGTGGCGGTCCCGGCGCTCGACGTTTCCCAGTCTGGCTCCGCACCTTGGCCCCCACTGTGGCGCGAGTTGGCGCCTACTTGG<br>CGGCGCGCTCAAGCCACCCCCTGGAGCCGGGCCGCCGCGCCCTGGACCCGGGAACGGACGTGGCCGAAGGCCAGGAGCACGGGAATGTCG<br>GTGAGGACGGGCAGATGGTAGGAGAACGGACGGGGGCCGACCATGTCGGGCGGGCCGCCGTAGCCGCCGTCCGCCTGCTGCCGTGCCAGC<br>AGCCAGGACAGGGCGCGCCCGACGGGGCGGGCCTCGGCCCCGTAGCACAGCGTGATCAG
**P*ermE**::*mfqS*-_dsOligo**	CTCGGCGGGTCTGGTGCTGGCCGGCACCTTCGCGGCGATGGCCTCGCTGCCGCTGGTGTTCGCGGCCGAACTCGGTTTCGCGGTGGCGTT<br>CGGCGTACTGCTGGACACCATGATCGTCCGCTCGGTGCTGGTCACCGCGCTGACGCTGGACGCGGACCGCTGGATGTGGTGGCCCAGCGC<br>GCTGTTCAGGCGTCAGGACGTCCCCCCGCCGCTGGACGAGGACAAGCGCGACCTCGAACCCGCCGTCGGGGGCAGGTGACCGACCCGGCT<br>TACTGACGGTGGCCCCCACGCACGCGATCAGCCGTGCGTGGGGGGCCACCGTCCGTGTGTGGCCCGAAGCCGCGGGAGTAATCCTGGGAT<br>TACTCGCCCGGGGTCGGCCCCGCCGGCACTTCGTGCAGGCGGTACCAGCCCGACCCGAGCACGCGCCGGCACGCCTGGTCGATGTCGGAC<br>CGGAGTTCGAGGTACGCGGCTTGCAGGTCCAGGAAGGGGACGTCCATGCGAGTGTCCGTTCGAGTGGCGGCTTGCGCCCGATGCTAGTCG<br>CGGTTGATCGGCGATCGCAGGTGCACGCGGTCGATCTTGACGGCTGGCGAGAGGTGCGGGGAGGATCTGACCGACGCGGTCCACACGTGG<br>CACCGCGATGCTGTTGTGGGCTGGACAATCGTGCCGGTTGGTAGGATCGGGCCCCCCCTCGAGGTCGACGGTATCGATAAGCTTGATATC<br>GAATTCCTGCAGCCCGGGGGATCCACTAGTTCTAGAGCGGCCCAGTGGAAAGTGAGGCGGCTCATGTCCGACGACACACAGGTACGCGAG<br>CACAACCGCGCGGTCGTCGCGCGGTACATGAACACCCGTGGCCAGGATCGCCTCGAGCGTCACCAGCTGTTCACGGAGGACGGCACCGGC<br>GGTCTGTGGACCACCGAGACCGGCGAACCCATCGTCATCAGTGGCCGGGACACCCTCGGCGAGCACGCCGTCTGGTCGCTGAAGTGCTTC<br>CCGGACTGGAAGTGGATCAACGTCGAGATCTTCGACACGCAGGACCCCGACCGGTTCTGGGTGGAGTGCGACGGCGAGGGCCAGATCCTG<br>TTCCCGGGCTATCCGGACGGTCACTACCGCAACCACTTCCTGCACTCGTTCCTGTTCGAGAACGGCAAGATCAAGCAGCA
**P*ermE**::*mfqM*-_dsOligo**	ACGTCGTCGGGATGGTAGTAGCAACGGCCGGCGTCGGTGTAGCGGCGCCATCGGTACTGGTGTGCGTATCGGTACACCGAGCCTTTGGAG<br>ACCCGCAGTAGGCCGACGATGTCATCCGCTGTCAGGCCCGCTGTCGCGGGCTCCCGATTCATATTCCTTCGCCCGGGTATTGAGGTGTGC<br>TCAAAATTCCCCCTTCGCGCGTGGATCCACCCACAACCCCCCGGATGCGAAACGCTGGTGGACCCGCCTGAGGGAGAGGTTCACCGAGAC<br>GGGCGGCACCCGTCAAGGGTCCCGGCGAGGGGGTGAACCGGCCCGTCCAACCCCCTTCTCCACAACCGGAACAAAAGCACGTTCGCATGG<br>ATCAAGGTCCCTCACTCTGCCGCTGTCGCCGGTTTCGCGGCAACGACGAGTTCAACGAGCAGGACGGGAAGCCGCGGGAGTAATCCTGGG<br>ATTACTCGCCCGGGGTCGGCCCCGCCGGCACTTCGTGCAGGCGGTACCGGGGGGGGGTCGAGGTCCAGCCCGACCCGAGCACGCGCCGGC<br>ACGCCTGGTCGATGTCGGACCGGAGTTCGAGGTACGCGGCTTGCAGGTCCAGGAAGGGGACGTCCATGCGAGTGTCCGTTCGAGTGGCGG<br>CTTGCGCCCGATGCTAGTCGCGGTTGATCGGCGATCGCAGGTGCACGCGGTCGATCTTGACGGCTGGCGAGAGGTGCGGGGAGGATCTGA<br>CCGACGCGGTCCACACGTGGCACCGCGATGCTGTTGTGGGCACAATCGTGCCGGTTGGTAGGATCGACGGTATCGATAAGCTTGATATCG<br>AATTCCTGCAGCCCGGGGGATCCACTAGTTCTAGAGCGGCCGTGGTCCGCATTCCCTTGGAGGCTTGAATGCCCGTAGCCACTCAATTGG<br>AAGACCTGTACGCGGCGATCGAGGAAACGGCTCAGCTGGCGCGCGTGGCCTGCTCGCGAGAGAAGGTCTGGCCTGTCCTGGACGCATTCG<br>GGGACGGGCTCGCGGACGCCCACGTCGTATTCAGCCTGGCGACCGGCGAACGGTACGCGGAAGAGCTCGCGTTCGATTTCACCGTGCCCC<br>CGGATGCGGGCGATCCGTATGCCGTCGCCGTGTCGAACGGACTCGTCGAGAAGACGGACCATCCGGTCGGCACCCTGTTCCCGGAGATCC<br>AGAGGCGCTGCCCCGTCGACAGCTTCGGTGTCGACTACGGAATCGTCG

Method S1. Synthesis of 2-*O*-methylflaviolin.

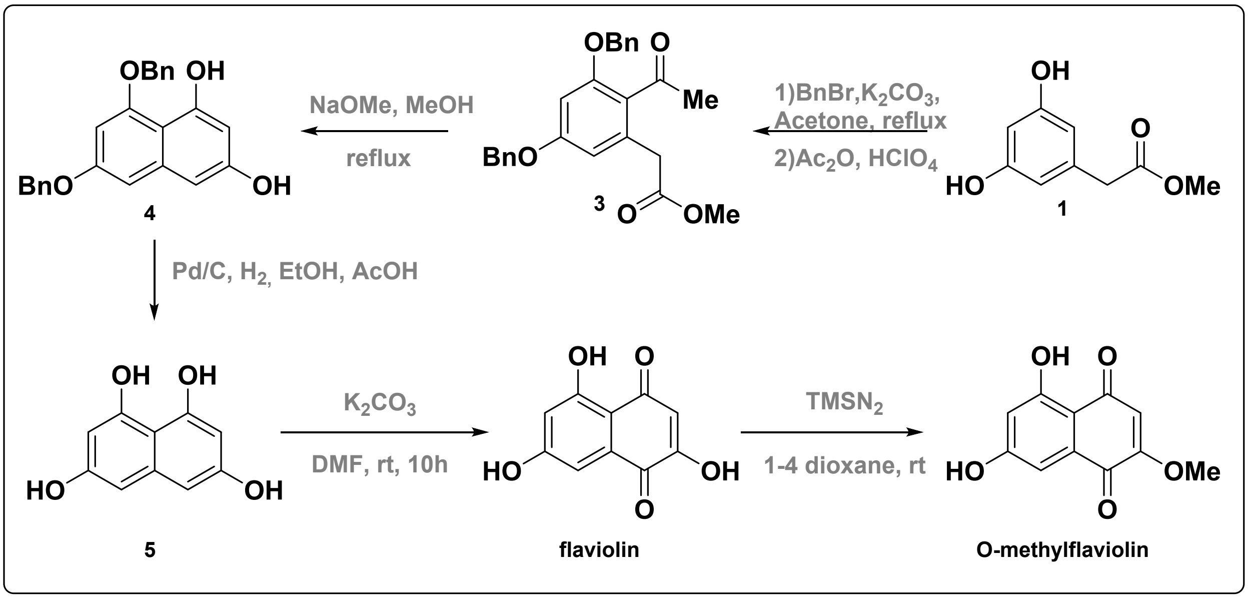

Spectral and Characterization Data

Methyl-[3,5-bis(benzyloxy)phenyl]acetate (2)[8]

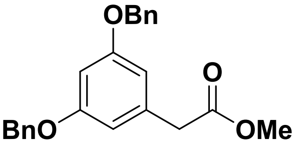

To a solution of methyl-3,5- dihydroxyphenylacetate (1) (500 mg, 2.7 mmol, 1 equiv) and benzyl bromide (0.68 mL, 5.8 mmol, 2.1 equiv) in acetone (30 mL), K_2CO_3 (933 mg, 6.7 mmol, 2.5 equiv) was added. The suspension was refluxed for 12 h, then filtered and washed with acetone. The filtrate was concentrated and the resulting solid was dissolved in EtOAc, washed with saturated (aq.) NaCl (10 mL), dried over anhydrous Na_2SO_4, filtered and concentrated under reduced pressure to give compound 2 in 90% (880 mg) as colorless leaves after crystalizzation from EtOAc/hexanes. All spectral data matched literature values.

^{1}H NMR (400MHz, $CDCl_3$): δ 7.44–7.34 (m, 10H, Bn H-2,2′,3,3′,4,4′,5,5′,6,6′), 6.56 (s, 3H, Ph H-2,4,6), 5.03 (s, 4H, Bn), 3.69 (s, 3H, CH_3), 3.57 (s, 2H, CH_2).
^{13}C NMR (100 MHz, $CDCl_3$): δ 171.9, 160.1, 136.9, 136.2, 128.7, 128.1, 127.7, 108.6, 100.9, 70.2, 52.2, 41.6.
HRMS (ESI): m/z calcd for $C_{23}H_{23}O_4{}^+$: 363.1596 $[M+H]^+$; found: 363.1591.

Methyl-[2-Acetyl-3,5-bis(benzyloxy)phenyl]acetate (3)[9]

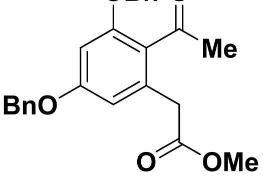

To a solution of methyl-[3,5-bis(benzyloxy)phenyl] acetate **(2)** (880 mg, 2.4 mmol, 1 equiv) in Ac_2O (18 ml), $HClO_4$ 70% (0.11 ml) was added dropwise. The resulting mixture was allowed

to react for 14 h at room temperature. The organic phase was extracted with EtOAc (2 × 100 mL), washed with saturated (*aq.*) NaCl (10 mL), dried over anhydrous Na_2SO_4, filtered and concentrated under reduced pressure. The resulting dark green residue was filtered through a short plug of silica gel (6:4, hexanes:EtOAc) and crystalized to give compound **3** in 85% yield (835 mg) as a fluffy white powder. All spectral data matched literature values.

^{1}H NMR (400MHz, $CDCl_3$): δ 7.35 (m, 10H, Bn H-2,2′,3,3′,4,4′,5,5′,6,6′) 6.52 (d, J = 1.6 Hz, 1H, Ph H-4), 6.44 (d, J = 1.6 Hz, 1H, Ph H-6), 5.05 (s, 2H, $BnCH_2$), 5.03 (s, 2H, $BnCH_2$), 3.72 (s, 2H, CH_2), 3.65 (s, 3H, CH_3), 2.48 (s, 3H, CH_3).
^{13}C NMR (100 MHz, $CDCl_3$): δ 199.8, 171.3, 166.5, 161.5, 141.2, 141.2, 137.9, 129.1, 129.1, 129.0, 129.0, 127.8, 127.7, 127.6, 127.6, 127.3, 127.2, 110.6, 106.0, 99.1, 74.3, 70.9, 51.9, 39.6, 29.9.
HRFAB (ES+): Calculated exact mass for $C_{25}H_{24}O_5$ [M] 404.16237. Found $[MH]^+$ 405.16215.

6,8-Bis-benzyloxynaphthalene-1,3-diol (4)[9].

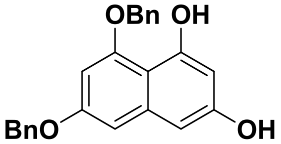

To the compound **(3)** (835 mg, 2.1 mmol, 1 equiv), a solution of sodium methoxide 0.5 M in MeOH (41 ml, 20.6 mmol, 10 equiv) in MeOH (50 mL) was added, then set to reflux for 45 min. The resulting mixture was acidified (5% HCl), extracted with EtOAc, concentrated under reduced pressure, filtered through Celite (dichloromethane) to give compound **4** in 80 % yield (625 mg) as white leaves after crystallization from dichloromethane/hexanes. All spectral data matched literature values.

^{1}H NMR (400MHz, $CDCl_3$): δ 9.32 (s, 1H, OH) 7.46 (m, 10H, Bn H-2,2′,3,3′,4,4′,5,5′,6,6′), 6.66 (d, J = 2 Hz, 1H, Ph H-4), 6.57 (d, J = 2.4 Hz, 1H, Ph H-5), 6.49 (d, J = 2 Hz, 1H, Ph H-7), 6.38 (d, J = 2.4 Hz, 1H, Ph H-2), 5.67 (bs, 1H, OH), 5.18 (s, 2H, $BnCH_2$), 5.10 (s, 2H, $BnCH_2$).
^{13}C NMR (100 MHz, $CDCl_3$): δ 158.9, 155.9, 153.2, 151.4, 141.3, 141.2, 137.4, 129.1, 129.1, 129.0, 129.0, 127.8, 127.7, 127.3, 127.3, 127.2, 127.2, 107.2, 100.9, 100.1, 97.2, 96.5, 71.3, 70.9.
HRFAB (ES+): Calculated exact mass for $C_{24}H_{20}O_4$ [M] 372.13616. Found [MH]+ 373.13647.

1,3,6,8-Tetrahydroxynaphthalene (5)[10]

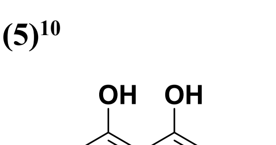

To compound **(4)** (620 mg, 1.6 mmol, 1 equiv), EtOH (2 mL) and AcOH (0.12 mL) under Argon atmosphere, palladium on carbon (10%, 1.7 mg) was added and the system was flushed with H_2, then allowed to react for 14 h. The suspension was filtered through Celite and recrystallized from acetone to give compound **5** in 65% yield (207 mg) as pale yellow solid. All spectral data matched literature values.

^{1}H NMR (400MHz, DMSO): δ 10.57 (br s, 2H, OH), 9.31 (s, 2H, OH), 6.28 (d, 2H, Ph H-4,5), 6.07 (d, 2H, Ph H-7,2).
^{13}C NMR (100 MHz, DMSO): δ 156.4, 155.4, 138.8, 104.2, 99.6, 97.8.

Flaviolin[11,12]

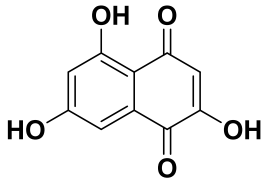

To a solution of naphthalene-1,3,6,8-tetraol (100 mg, 0.5 mmol, 1.0 equiv) in DMF (10 mL) K_2CO_3 (288 mg, 2.1 mmol, 4.0 equiv) was added. The mixture was stirred at 25 °C for 10 h, open to air. Upon completion, the reaction was slowly acidified with concentrated HCl until a pH of 2 was reached. The organic phase was extracted with Et_2O (2 × 100 mL), washed with saturated (*aq.*) NaCl (10 mL), dried over anhydrous Na_2SO_4, filtered and concentrated under reduced pressure to give **flaviolin** in 99% yield (106 mg) as a red solid.

^{1}H NMR (400MHz, acetone-d6): δ = 12.59 (s, 1H, OH), 9.85 (bs, 2H, PhOH), 7.10 (d, J = 2.4 Hz, 1H, Ph H-8), 6.63 (d, J = 2.4 Hz, 1H, Ph H-6), 6.13 (s, 1H, CH).
^{13}C NMR (100 MHz, acetone-d6): δ = 191.4, 181.8, 164.6, 164.5, 159.8, 133.2, 111.1, 109.5, 109.1, 108.9.
HRMS (FAB): calcd. for $C_{10}H_7O_5$: 207.0293 [M + H^+], found: 207.0292.

O-methylflaviolin[13]

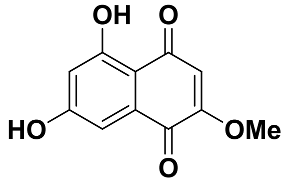

To a solution of flaviolin (100 mg, 0.5 mmol, 1.0 equiv) in dry 1-4 dioxane (10 mL) (Trimethylsilyl)diazomethane solution 2.0 M in diethyl ether (0.37 ml, 0.7 mmol, 1.5 equiv) was slowly dropwise at room temperature till the reaction was complete under Argon atmosphere. Upon completion, the reaction mixture was concentrated under reduced pressure and the solid was recrystallized from chloroform to give **O-methylflaviolin** in 98% yield (106 mg) as an orange solid.

^{1}H NMR (400 MHz, acetone-d6): δ = 12.47 (s, 1 H, α-OH), 9.80 (s, 1 H, β-OH), 7.07 (d, J = 2.1 Hz, 1 H, Ph H-8), 6.61 (d, J = 2.1 Hz, 1 H, Ph H-6), 6.15 (s, 1 H, CH), 3.93 (s, 3 H, OCH_3).
^{13}C NMR (100 MHz, acetone-d6): δ = 190.6, 179.7, 164.7, 164.6, 161.9, 134.1, 110.1, 108.99, 108.9, 108.7, 57.1.
HRMS (ESI), *m/z*: calcd. for $C_{11}H_7O_5$: 219.0299 $[M-H]^-$, found: 219.0295.

^{1}H-, ^{13}C-NMR Spectra for the Compounds

Flaviolin

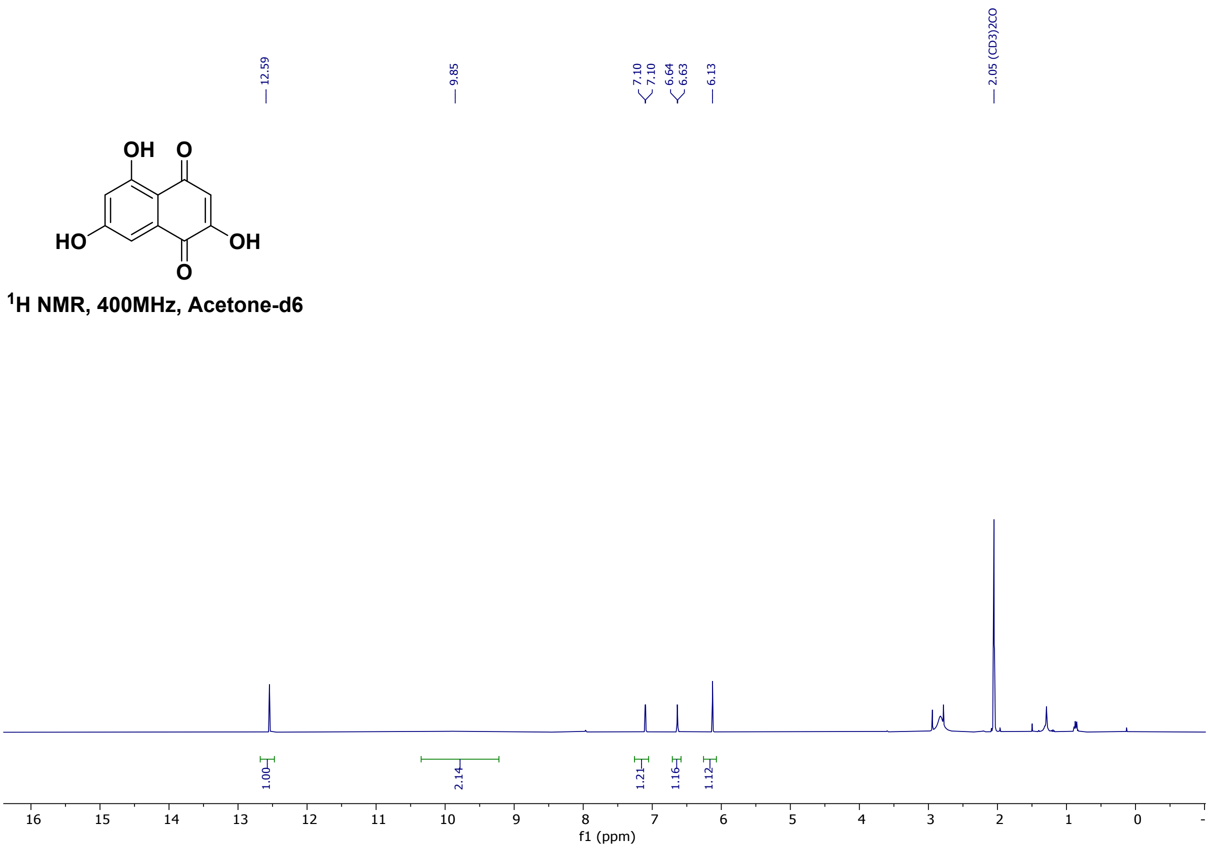

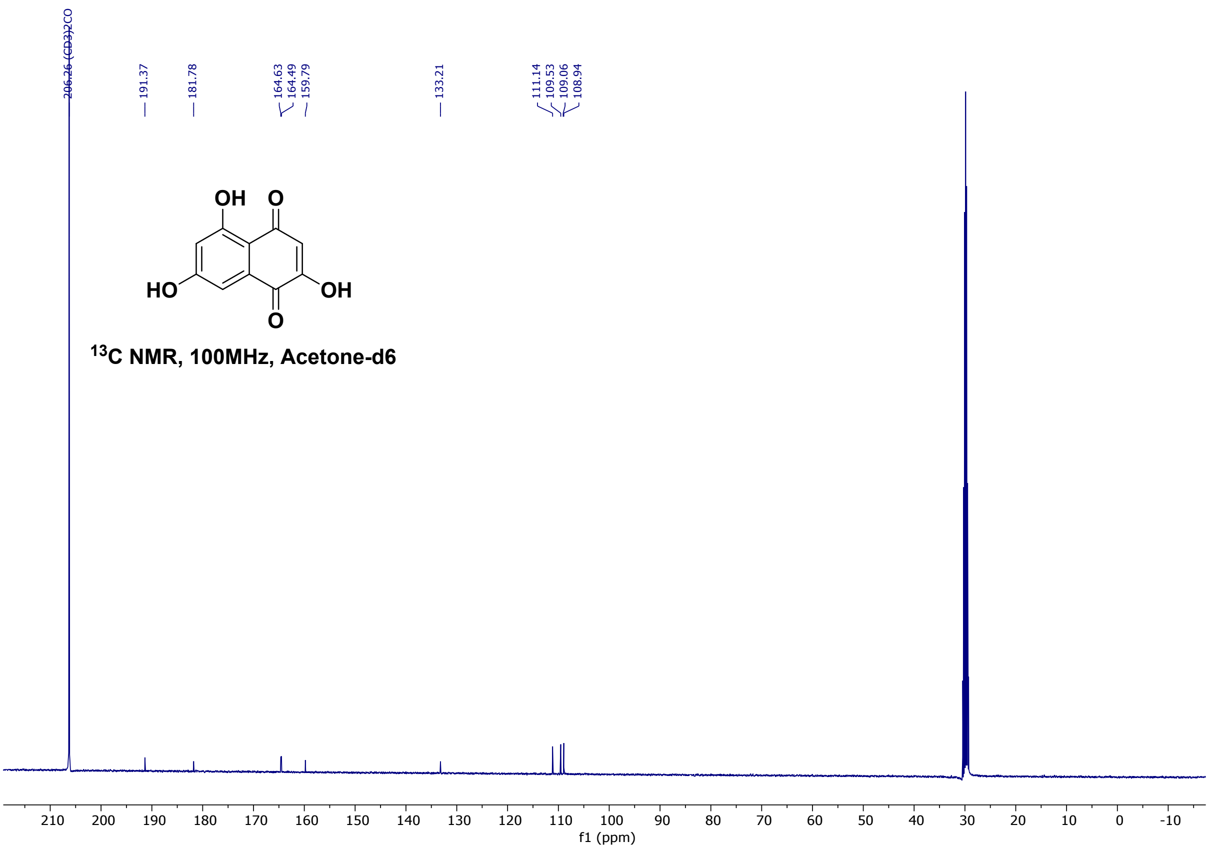

O-methylflaviolin

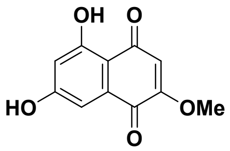

^{1}H NMR, 400MHz, Acetone-d6

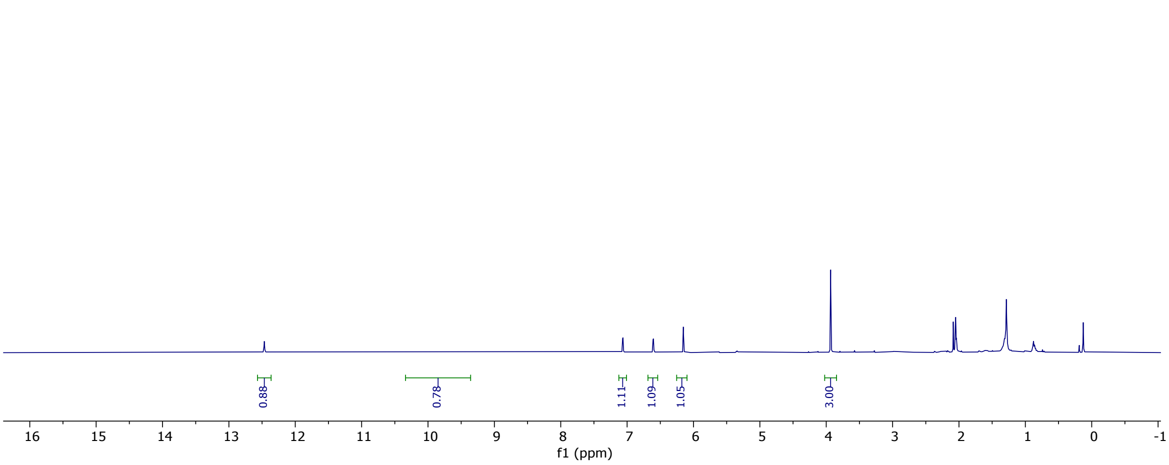

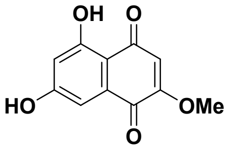

^{13}C NMR, 100MHz, Acetone-d6

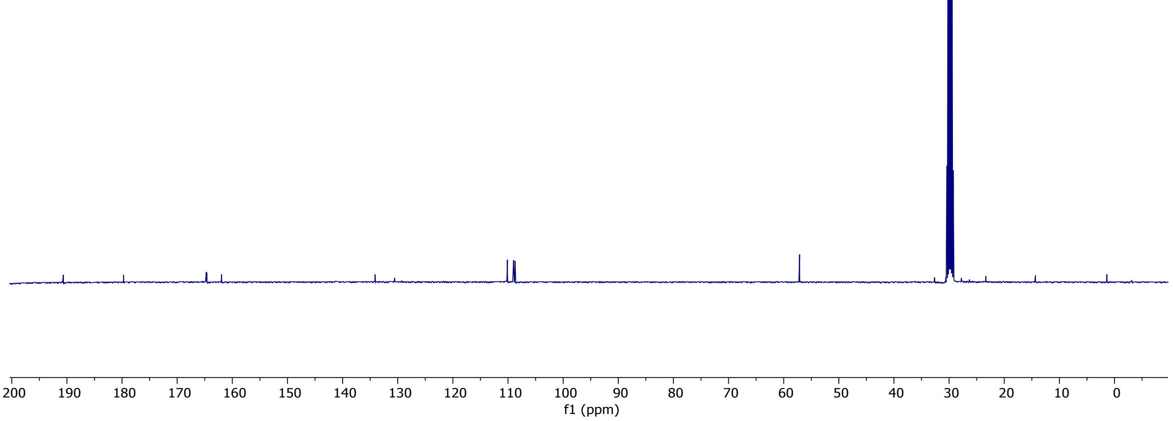

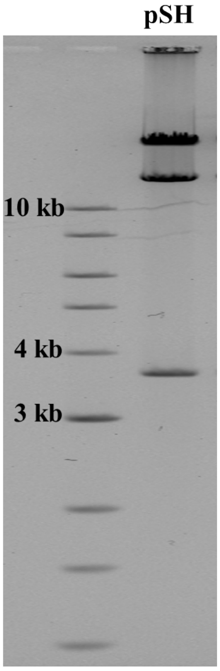

Figure S1. Restriction analysis of pCLY10::*mfq* following assembly in *Saccharomyces cerevisiae* BY4742 and cloning into *Escherichia coli* EPI300 using *Nde*I and *Hind*III (Expected Fragment Sizes: 3768, 15216, 27917 bp)

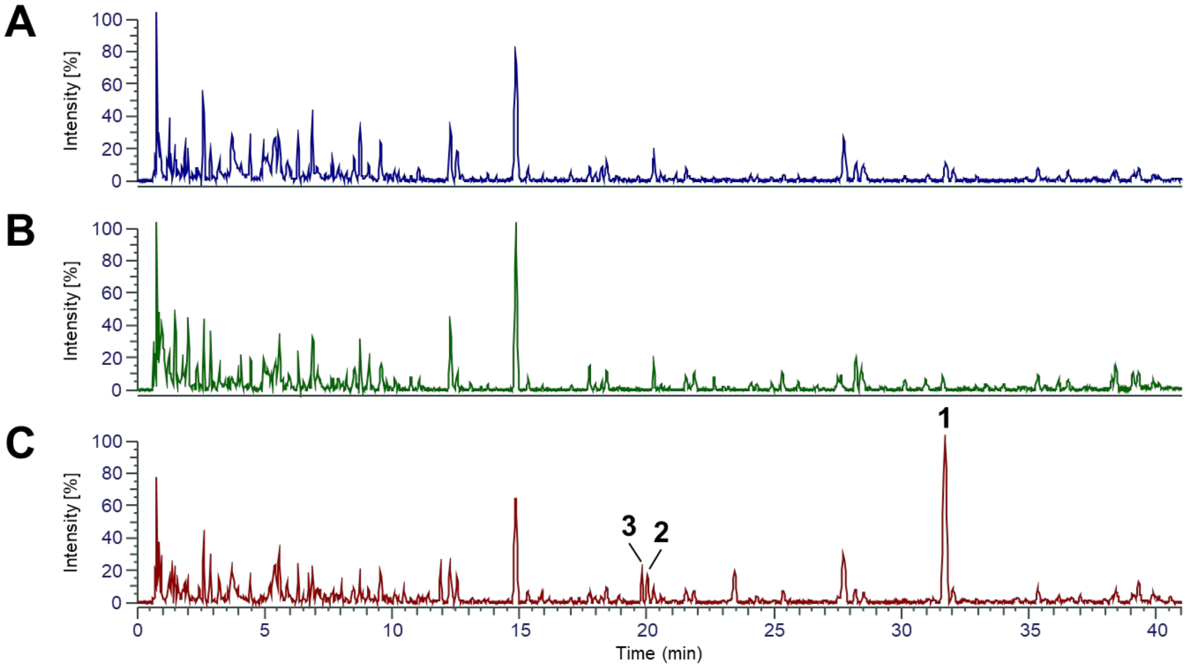

Figure S2. Base peak chromatograms (*m/z* 140-2000) obtained by LC-MS in positive ion mode of the acetone extracts from the pellets of strain *S. coelicolor* M1154 (A), strain *S. coelicolor* M1154 pCLY10/pUWLoriT (B), and strain *S. coelicolor* M1154 *pCLY10::mfq*/pOE_*mfqF* (C) grown in MYM medium. The peaks corresponding to marfuraquinocin E (**1**), marfuraquinocin C or D (**2**), and an undescribed congener with the sum formula $C_{26}H_{34}O_6$ (**3**) are labelled.

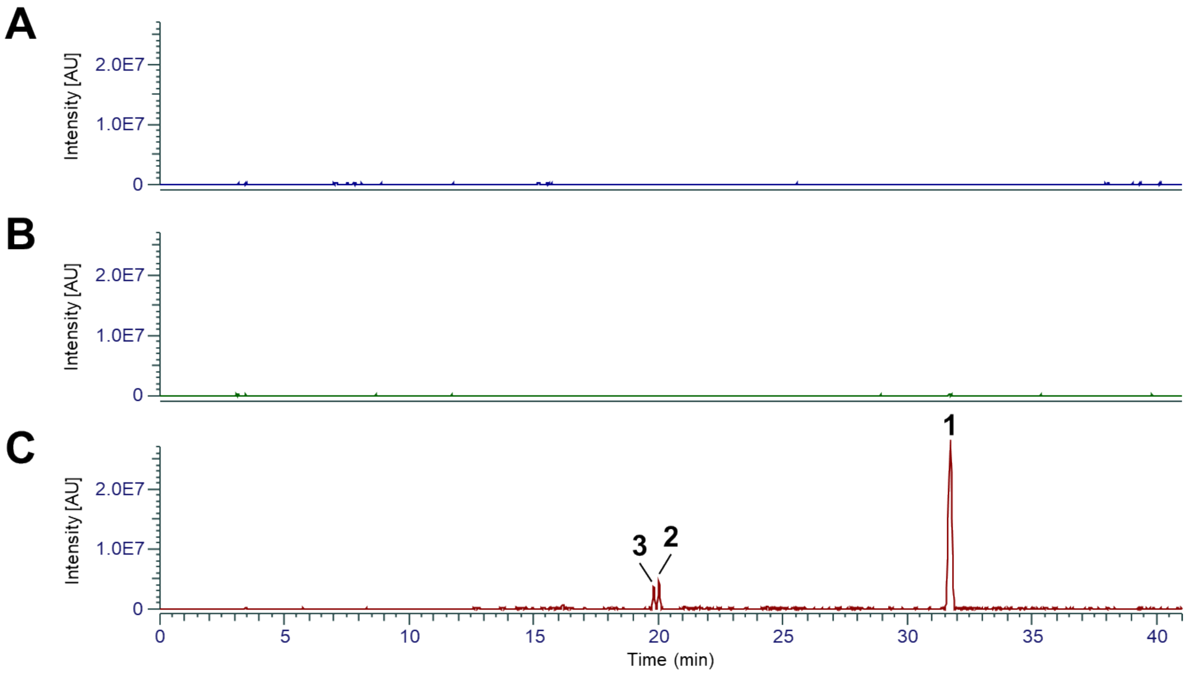

Figure S3. Extracted ion chromatograms (*m/z* 443.2428, 441.2272, and 427.2479, all ± 5ppm, corresponding to the $[M+H]^+$ ions of $C_{26}H_{34}O_6$, $C_{26}H_{32}O_6$, $C_{26}H_{34}O_5$, respectively) obtained by LC-MS in positive ion mode of the acetone extracts from the pellets of strain *S. coelicolor* M1154 (A), strain *S. coelicolor* M1154 pCLY10/pUWLoriT (B), and strain *S. coelicolor* M1154 *pCLY10::mfq*/pOE_*mfqF* (C) grown in MYM medium. The peaks corresponding to marfuraquinocin E (**1**), marfuraquinocin C or D (**2**), and an undescribed congener with the sum formula $C_{26}H_{34}O_6$ (**3**) are labelled.

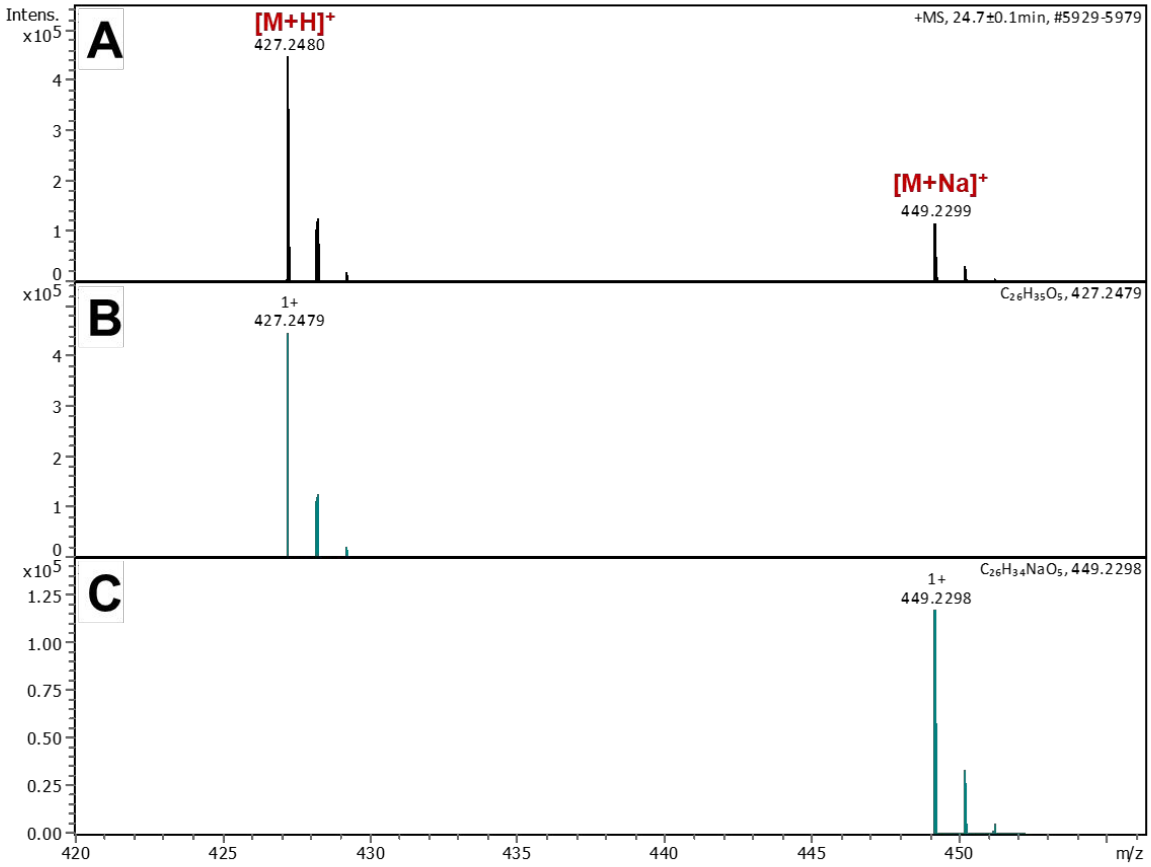

Figure S4. High resolution ESI-Qq-TOF mass spectrum of marfuraquinocin E (**1**) (A) and simulated isotopic patterns of the $[M+H]^+$ ion (B) and $[M+Na]^+$ ion (C) of a compound with the sum formula $C_{26}H_{34}O_5$.

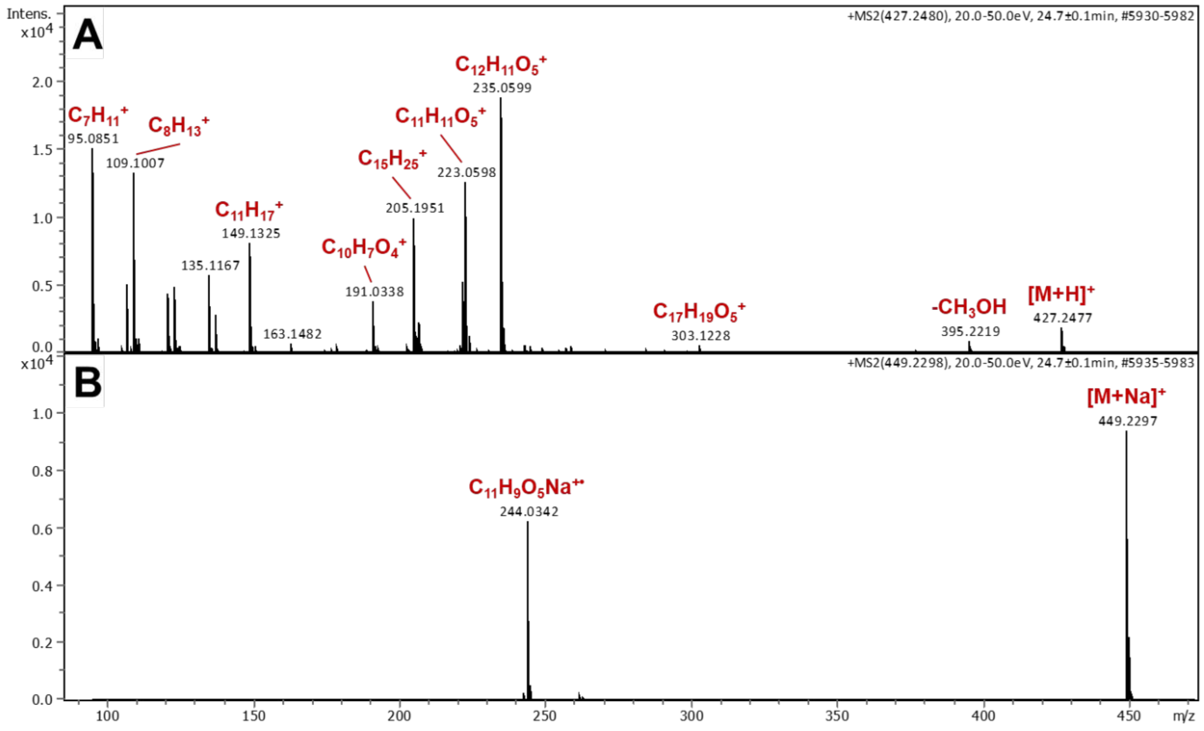

Figure S5. High resolution ESI-Qq-TOF MS/MS spectra of the $[M+H]^+$ ion (A) and $[M+Na]^+$ ion (B) of marfuraquinocin E (**1**).

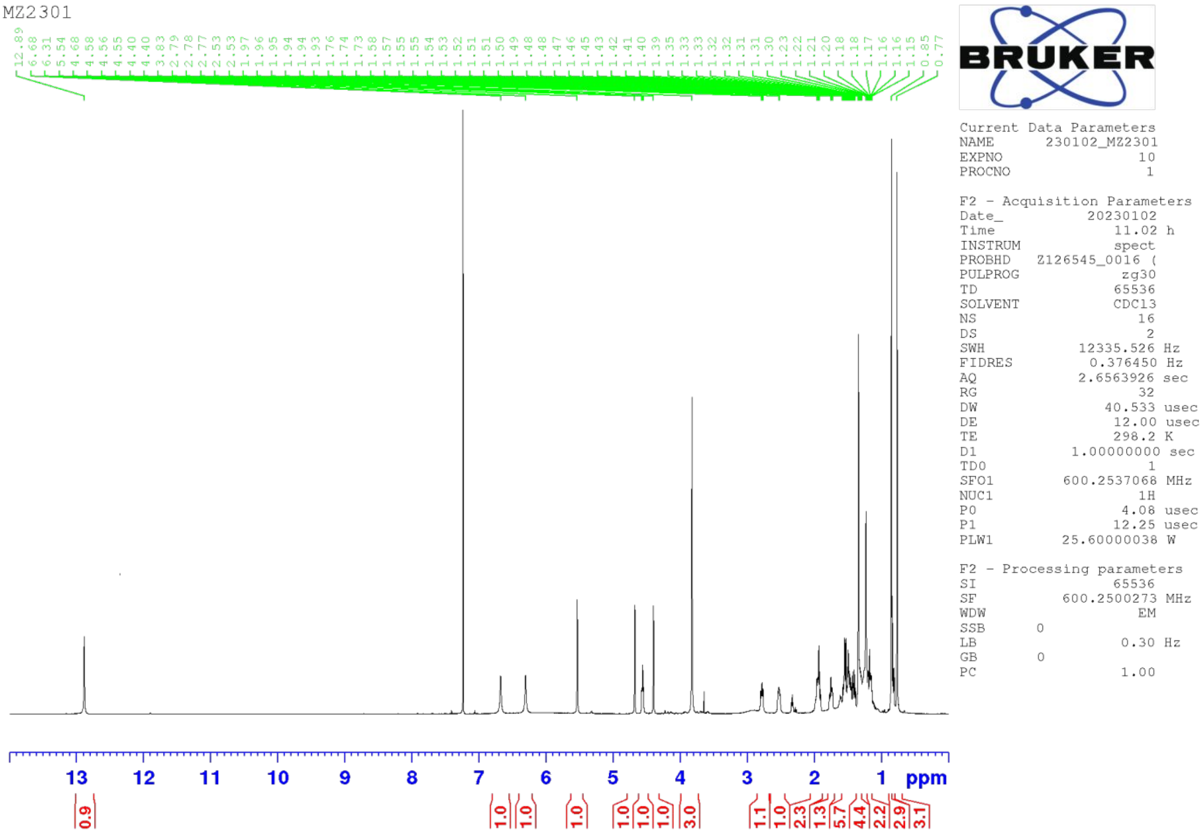

Figure S6. ^{1}H NMR spectrum of marfuraquinocin E (**1**) in $CDCl_3$ at 600 MHz.

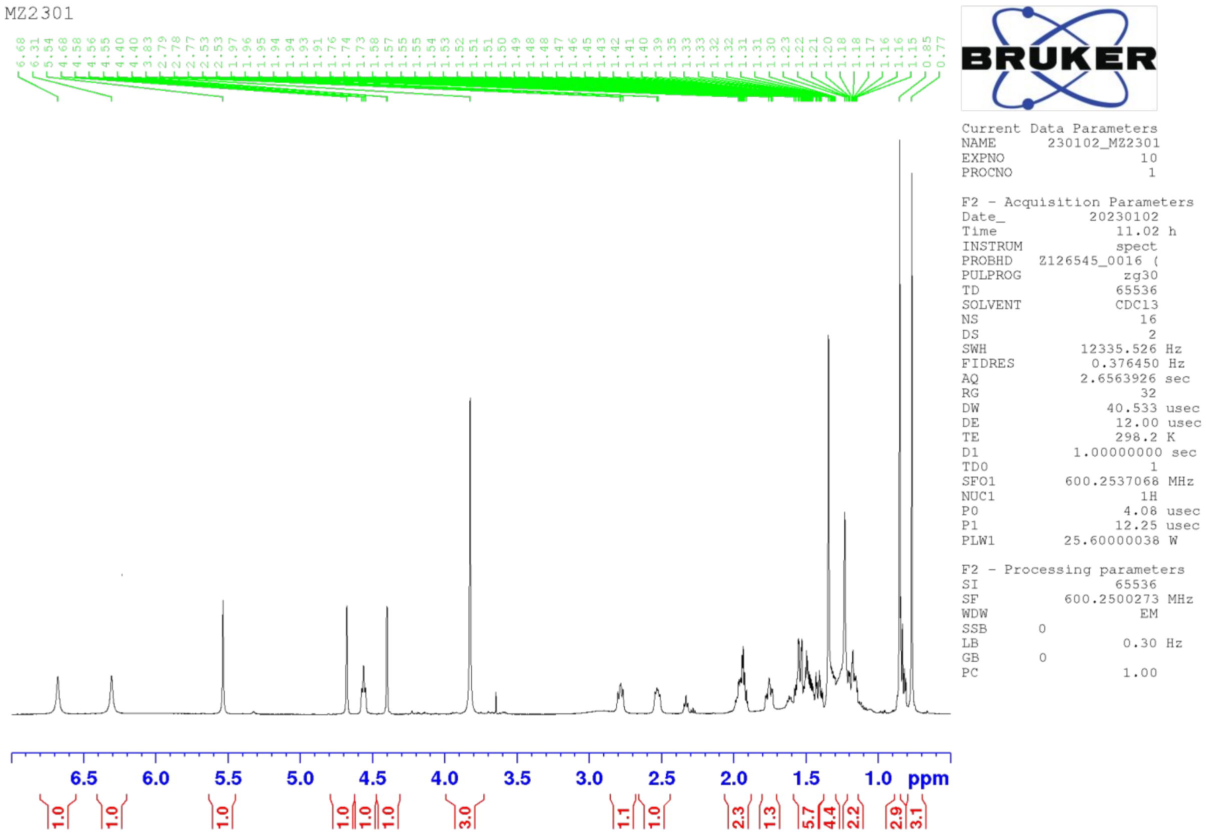

Figure S7. ^{1}H NMR spectrum of marfuraquinocin E (**1**) in $CDCl_3$ at 600 MHz (zoom).

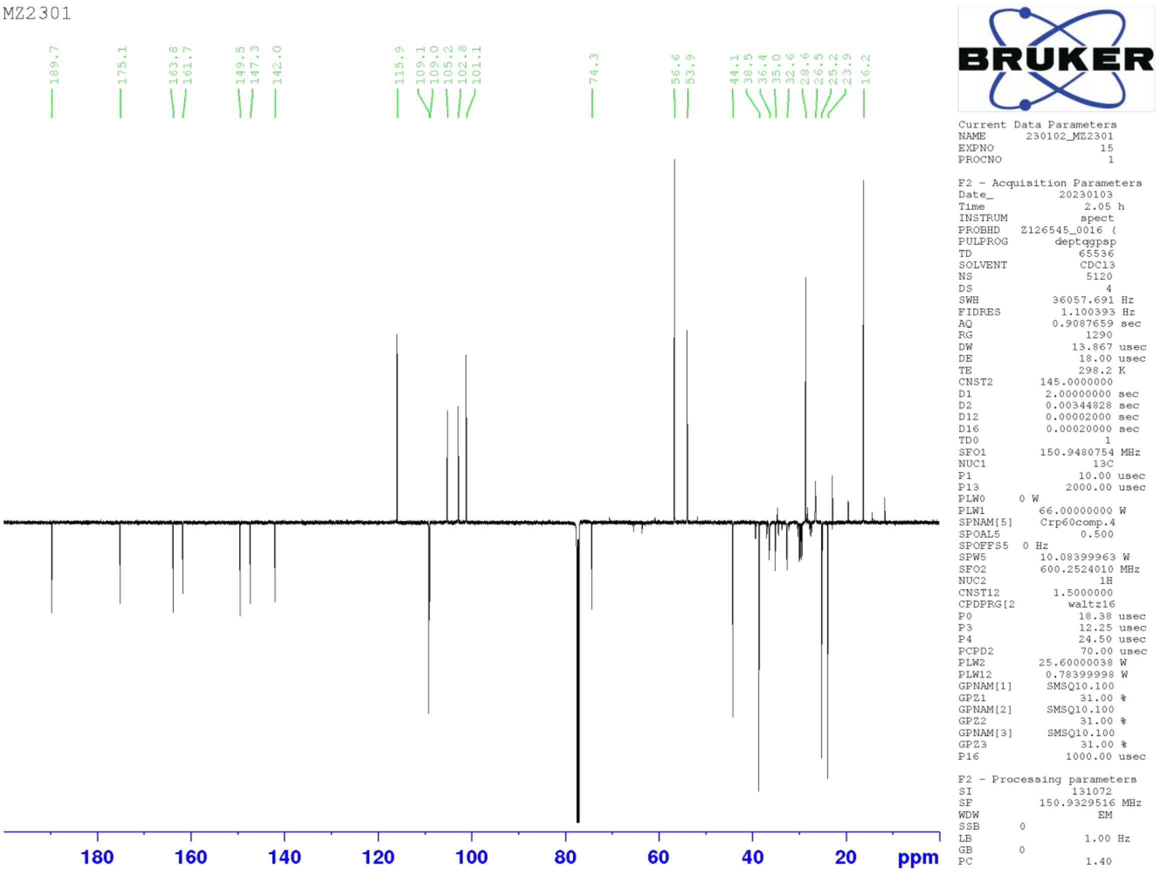

Figure S8. ^{13}C (DEPTq) NMR spectrum of marfuraquinocin E (**1**) in $CDCl_3$ at 600 MHz.

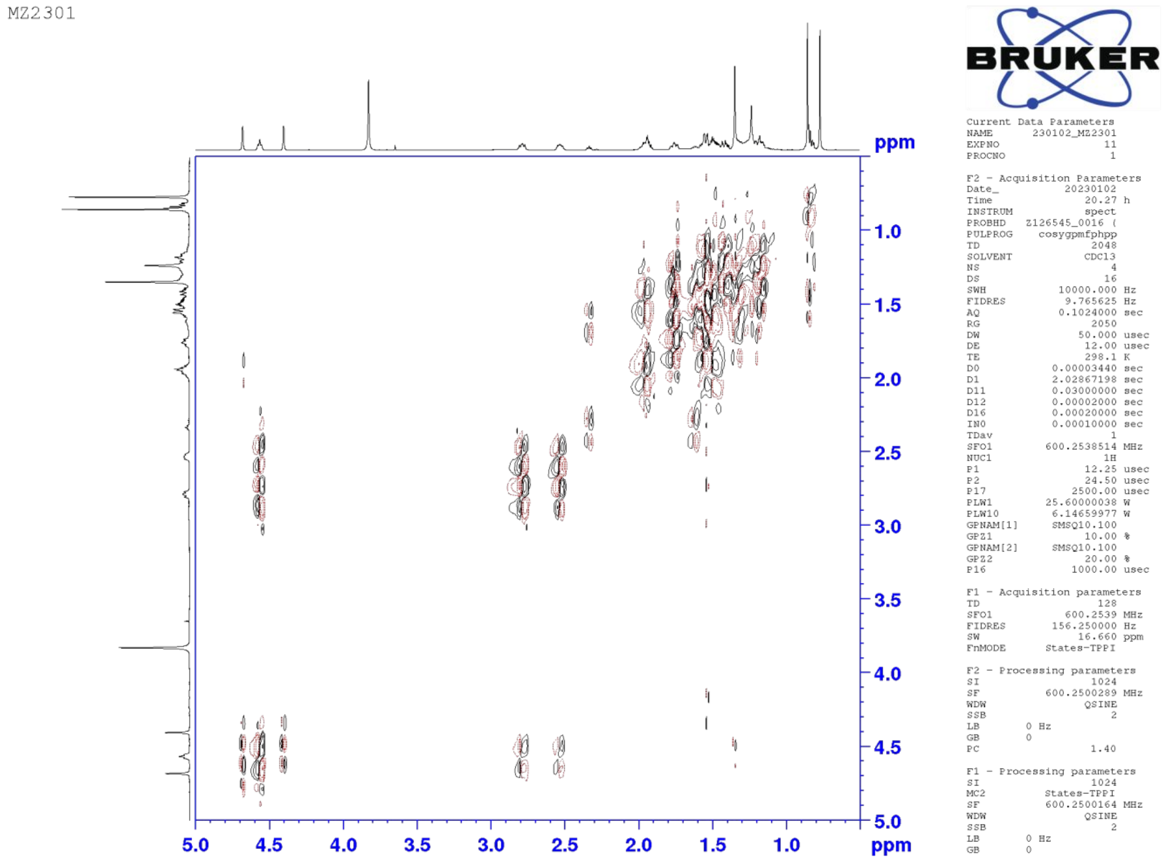

Figure S9. COSY spectrum of marfuraquinocin E (**1**) in $CDCl_3$ at 600 MHz.

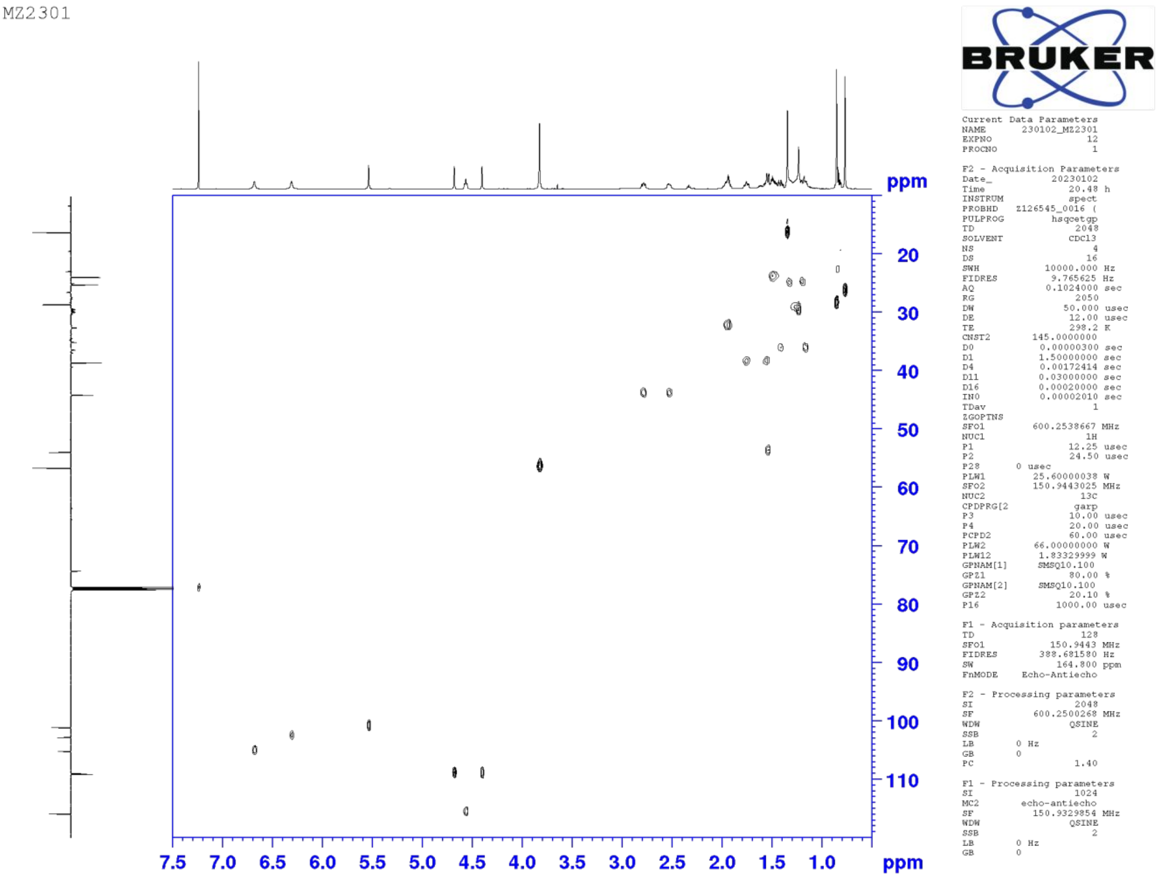

Figure S10. HSQC spectrum of marfuraquinocin E (**1**) in $CDCl_3$ at 600 MHz.

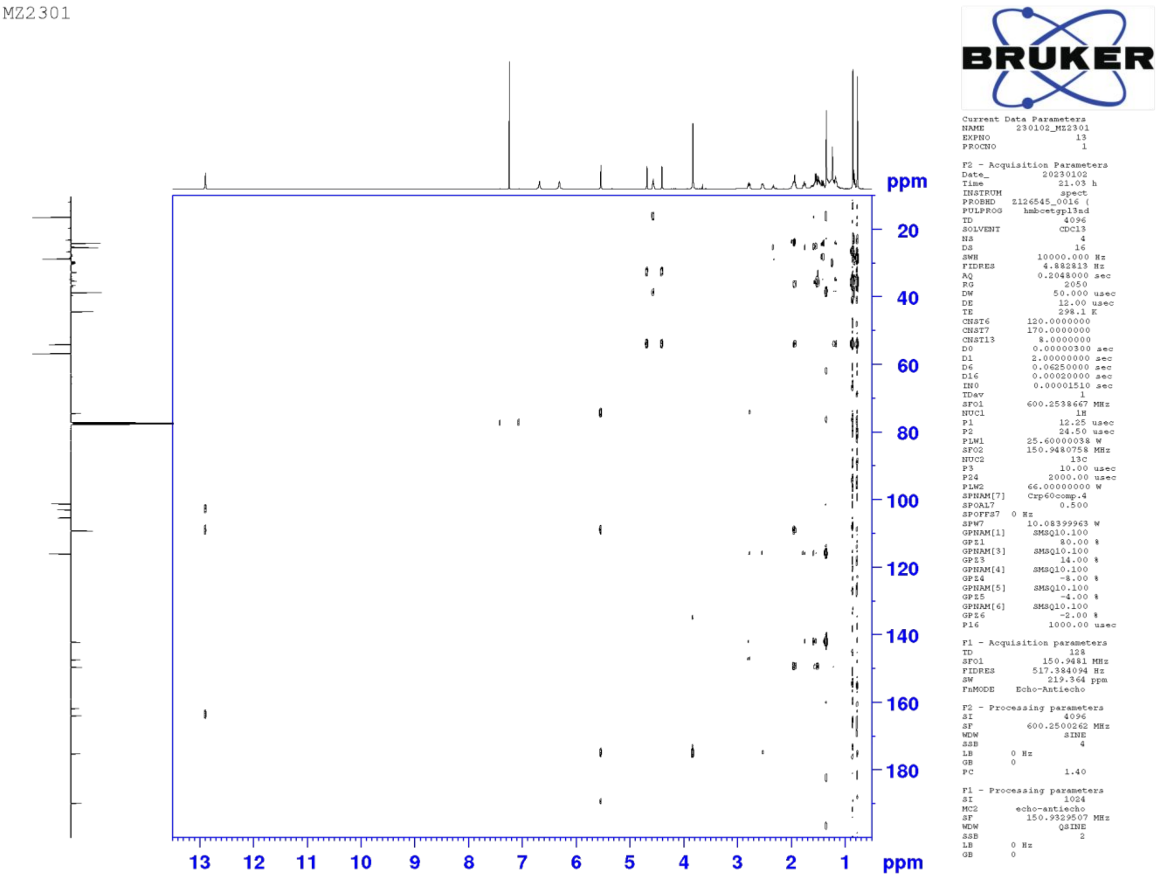

Figure S11. HMBC spectrum of marfuraquinocin E (**1**) in $CDCl_3$ at 600 MHz.

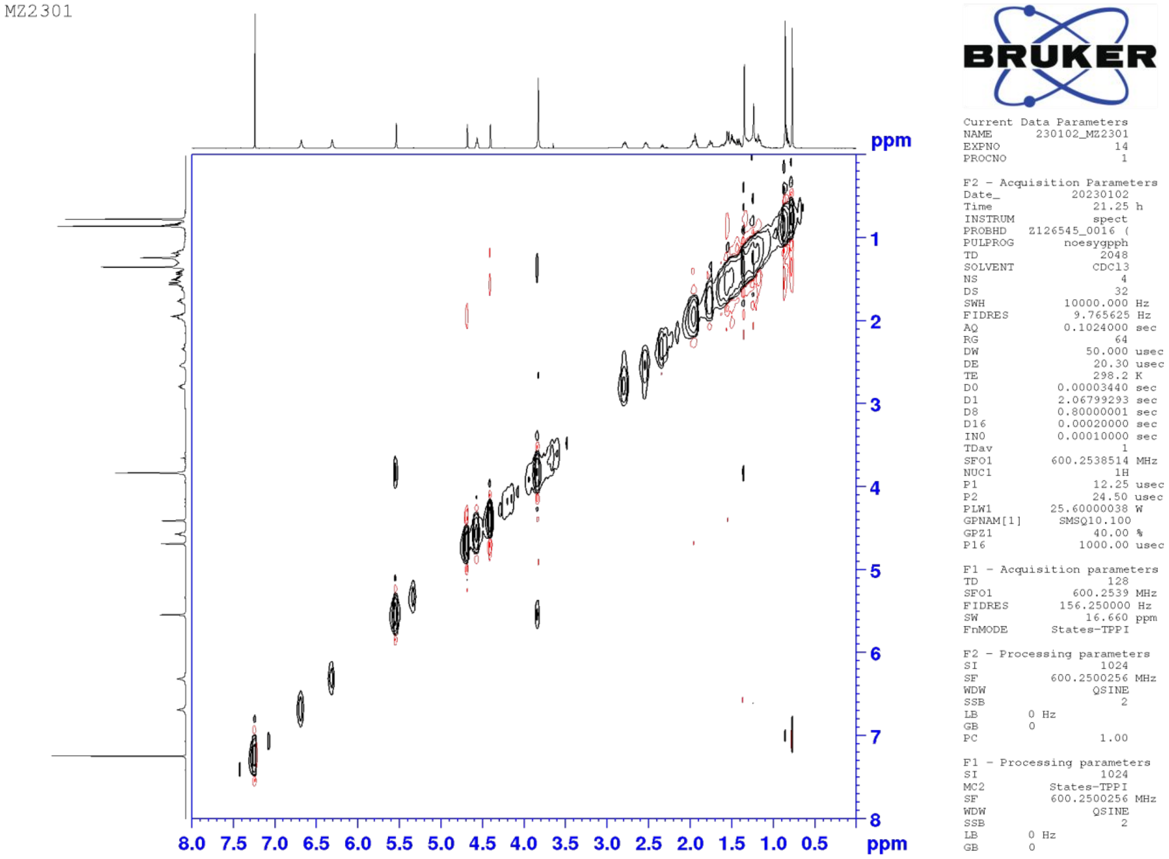

Figure S12. NOESY spectrum of marfuraquinocin E (**1**) in $CDCl_3$ at 600 MHz.

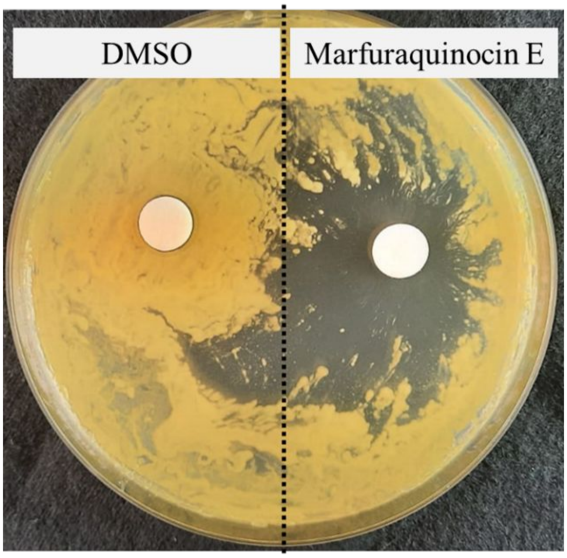

Figure S13. Disc diffusion assay demonstrating antibiotic susceptibility of *Micrococcus luteus* DSMZ 1790 to marfuraquinocin E.

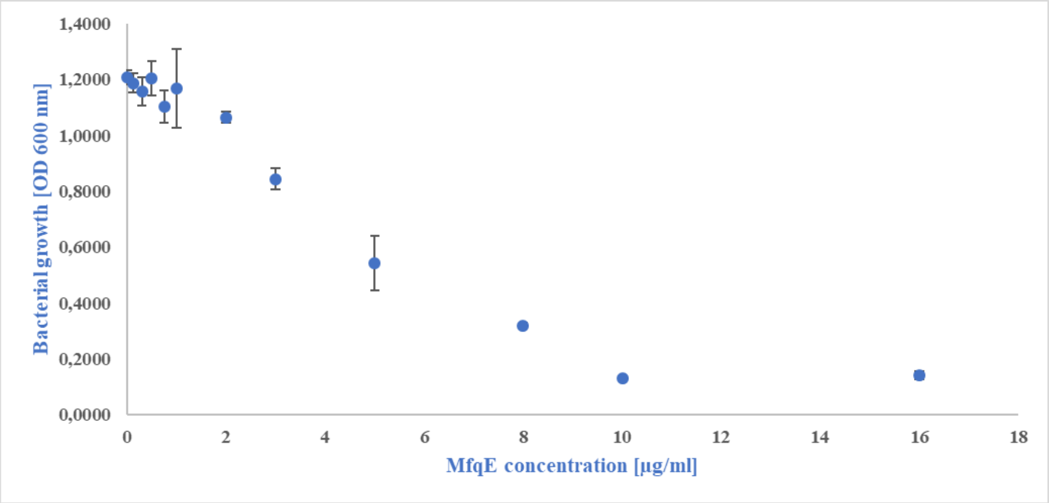

Figure S14. Determination of MIC of marfuraquinocin E (0–16 µg/mL) against *Micrococcus luteus* DSMZ 1790 in liquid TSB after 18 h incubation in a microtiter plate. Results represent the mean of triplicates.

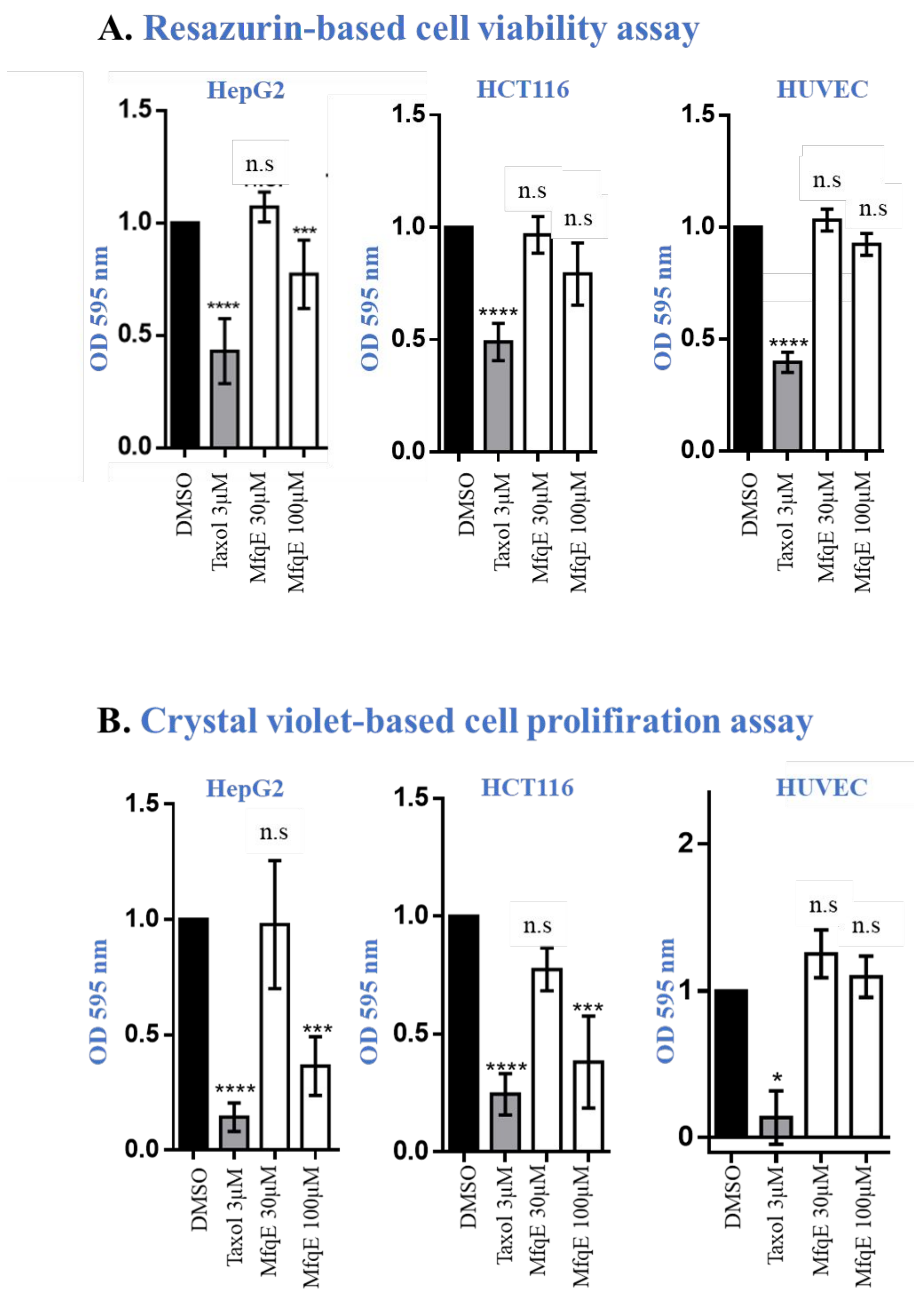

Figure S15. Assessment of cell viability (A) and proliferation (B) using resazurin and crystal violet assays. Optical density (OD 595 nm) normalized to vehicle control. Statistical analysis was performed using one-way ANOVA followed by Dunnett's post hoc test. ****$p < 0.0001$. Data are presented as mean ± SD from three independent experiments ($n = 3$) with quadruplicate wells. Cell lines: HepG2, HCT116, HUVEC.

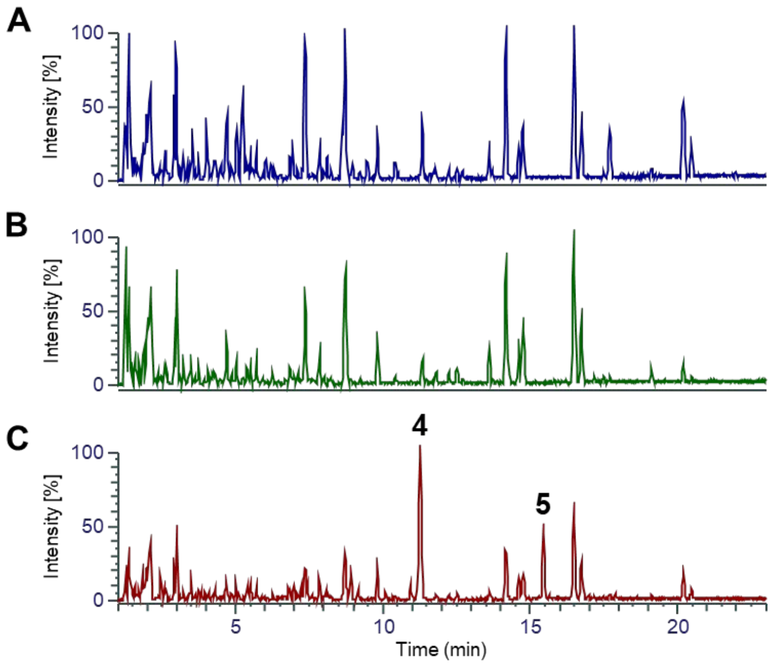

Figure S16. Base peak chromatograms (*m/z* 140-2000) obtained by LC-MS in negative ion mode of the acetone extracts from the pellets of the *S. coelicolor* M1154 strains carrying the plasmids pCLY10/pOE_*mfqF* (A), *pCLY10::mfq*Δ*mfqH*/pUWLoriT (B), and *pCLY10::mfq*Δ*mfqH*/pOE_*mfqF* (C) grown in MYM medium. The peaks corresponding to the tentatively identified flaviolin (**4**) and biflaviolin isomer (**5**) are labelled.

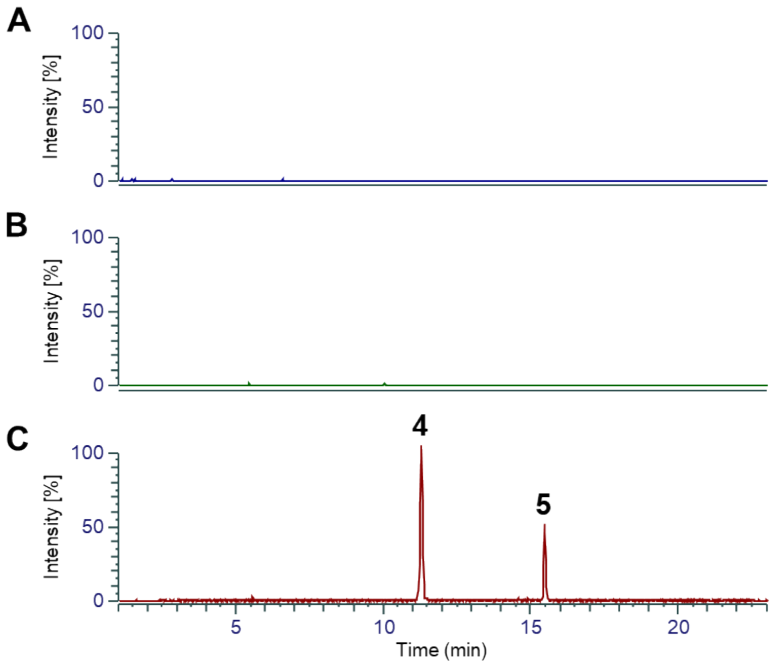

Figure S17. Extracted ion chromatograms (*m/z* 205.0142 and 409.0201, both ± 5ppm, corresponding to the $[M\text{-}H]^-$ ions of $C_{10}H_6O_5$ and $C_{20}H_{10}O_{10}$, respectively) obtained by LC-MS in negative ion mode of the acetone extracts from the pellets of the *S. coelicolor* M1154 strains carrying the plasmids pCLY10/pOE_*mfqF* (A), *pCLY10::mfq*Δ*mfqH*/pUWLoriT (B), and *pCLY10::mfq*Δ*mfqH*/pOE_*mfqF* (C) grown in MYM medium. The peaks corresponding to the tentatively identified flaviolin (**4**) and biflaviolin isomer (**5**) are labelled.

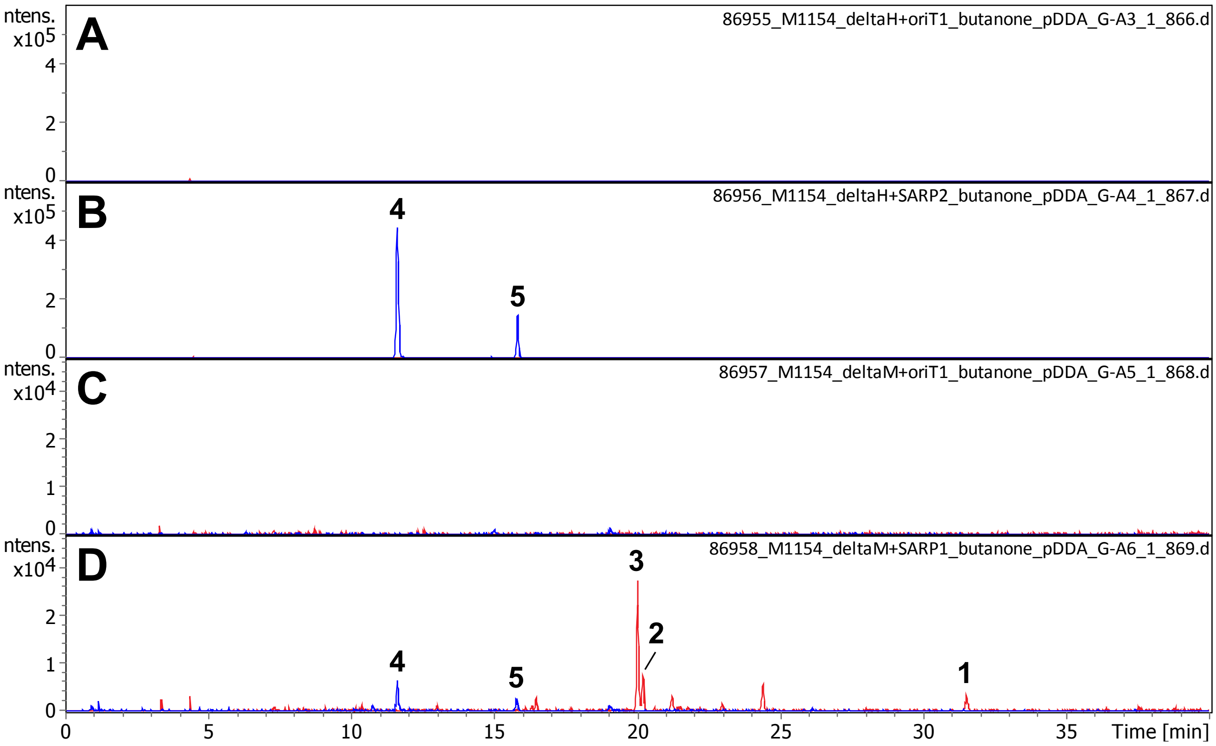

Figure S18. Extracted ion chromatograms (EICs) obtained by LC-MS in positive ion mode of the butanone extracts from the supernatants of the *S. coelicolor* M1154 strains carrying the plasmids *pCLY10::mfq*Δ*mfqH*/pUWLoriT (A), *pCLY10::mfq*Δ*mfqH*/pOE_*mfqF* (B), *pCLY10::mfq*Δ*mfqM*/pUWLoriT (C), and *pCLY10::mfq*Δ*mfqM*/pOE_*mfqF* (D) grown in MYM medium. The red trace shows the EIC of *m/z* 443.2428, 441.2272, and 427.2479 (all ± 5ppm) corresponding to the $[M+H]^+$ ions of $C_{26}H_{34}O_6$, $C_{26}H_{32}O_6$, and $C_{26}H_{34}O_5$, respectively. The blue trace shows the EIC of *m/z* 207.0288 and 411.0347 (all ± 5ppm) corresponding to the $[M+H]^+$ ions of $C_{10}H_6O_5$ and $C_{20}H_{10}O_{10}$, respectively. The peaks corresponding to marfuraquinocin E (**1**), marfuraquinocin C or D (**2**), an undescribed congener with the sum formula $C_{26}H_{34}O_6$ (**3**), flaviolin (**4**), and a biflaviolin isomer (**5**) are labelled.

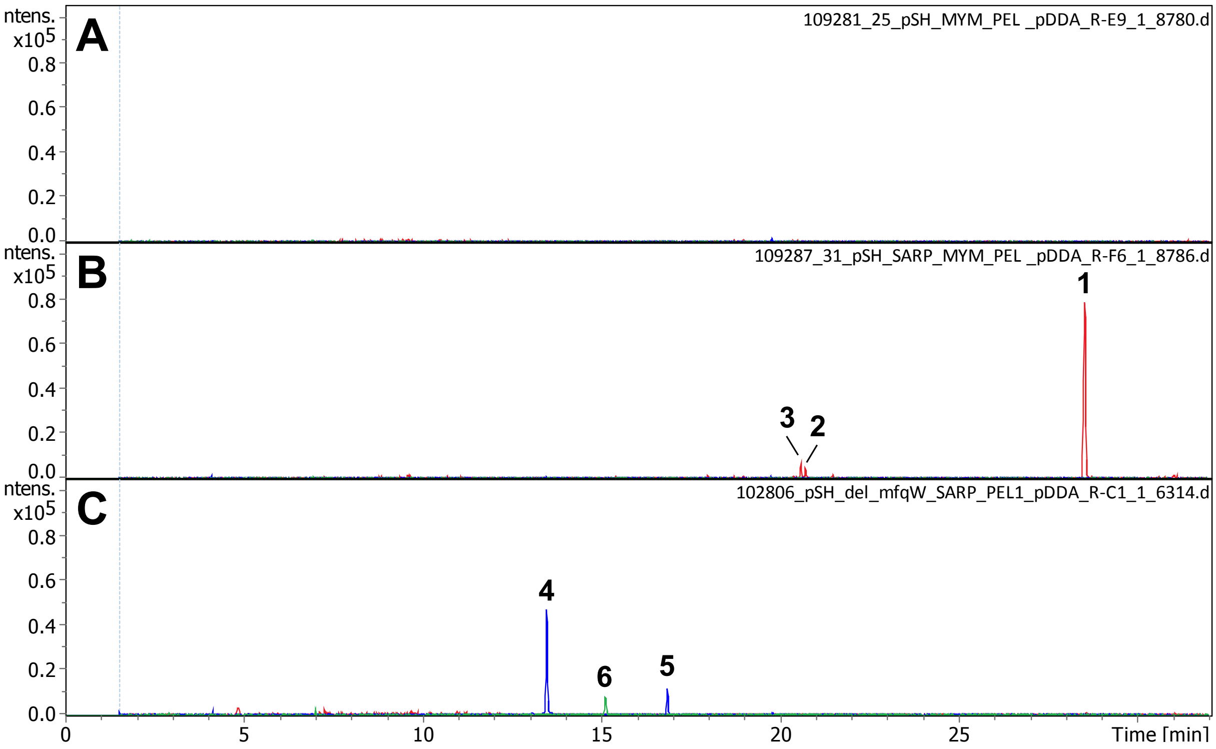

Figure S19. Extracted ion chromatograms (EICs) obtained by LC-MS in positive ion mode of the acetone extracts from the pellets of the *S. coelicolor* M1154 strains carrying the plasmids *pCLY10::mfq* (A), *pCLY10::mfq*/pOE_*mfqF* (B), and *pCLY10::mfq*Δ*mfqW*/pOE_*mfqF* (C) grown in MYM medium. The red trace shows the EIC of *m/z* 443.2428, 441.2272, and 427.2479 (all ± 5ppm) corresponding to the $[M+H]^+$ ions of $C_{26}H_{34}O_6$, $C_{26}H_{32}O_6$, and $C_{26}H_{34}O_5$, respectively. The blue trace shows the EIC of *m/z* 207.0288 and 411.0347 (all ± 5ppm) corresponding to the $[M+H]^+$ ions of $C_{10}H_6O_5$ and $C_{20}H_{10}O_{10}$, respectively. The green trace shows the EIC of *m/z* 221.0445 (± 5ppm) corresponding to the $[M+H]^+$ ion of $C_{11}H_8O_5$. The peaks corresponding to marfuraquinocin E (**1**), marfuraquinocin C or D (**2**), an undescribed congener with the sum formula $C_{26}H_{34}O_6$ (**3**), flaviolin (**4**), a biflaviolin isomer (**5**), and 2-*O*-methylflaviolin (**6**) are labelled.

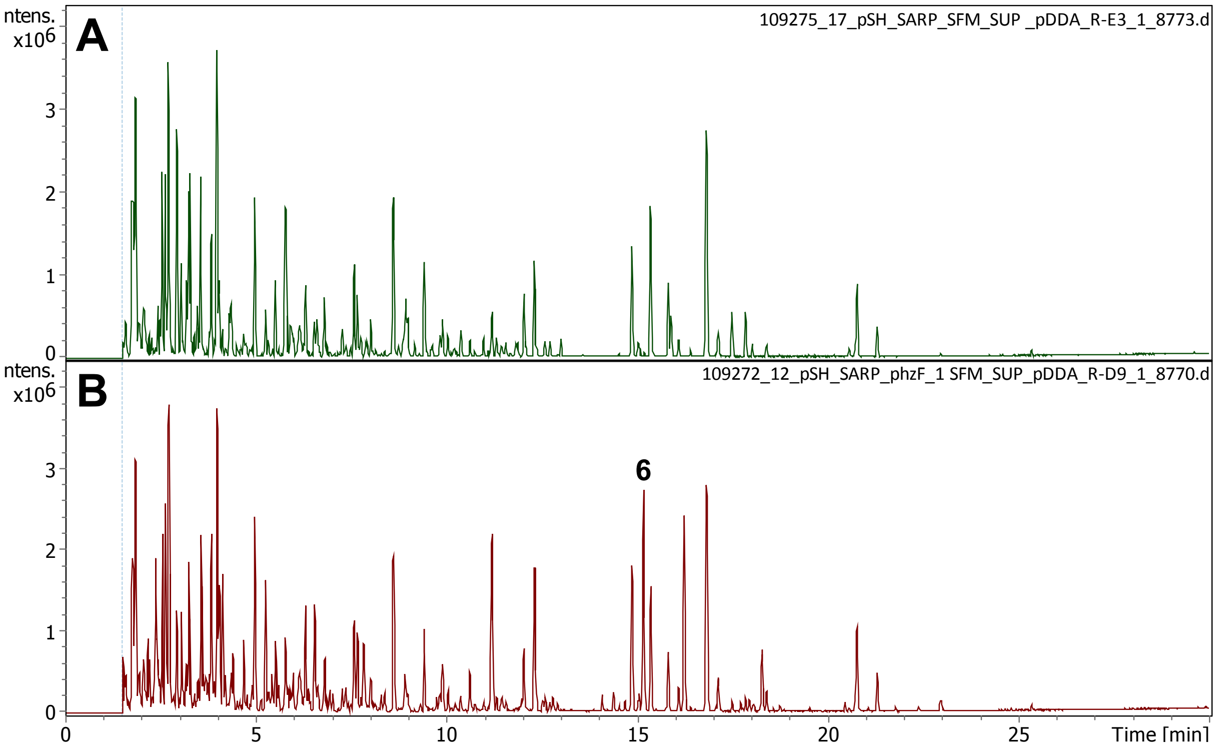

Figure S20. Base peak chromatograms (*m/z* 100-2500) obtained by LC-MS in positive ion mode of the methanol extracts from the supernatant of the *S. coelicolor* M1154 strains carrying the plasmids *pCLY10::mfq*/pOE_*mfqF* (A) and *pCLY10::mfq*/pOE_*phzF-mfqF* (B) grown in SFM medium. The peak corresponding to the tentatively identified phenazine-1,6-dicarboxylic acid (**6**) is labelled.

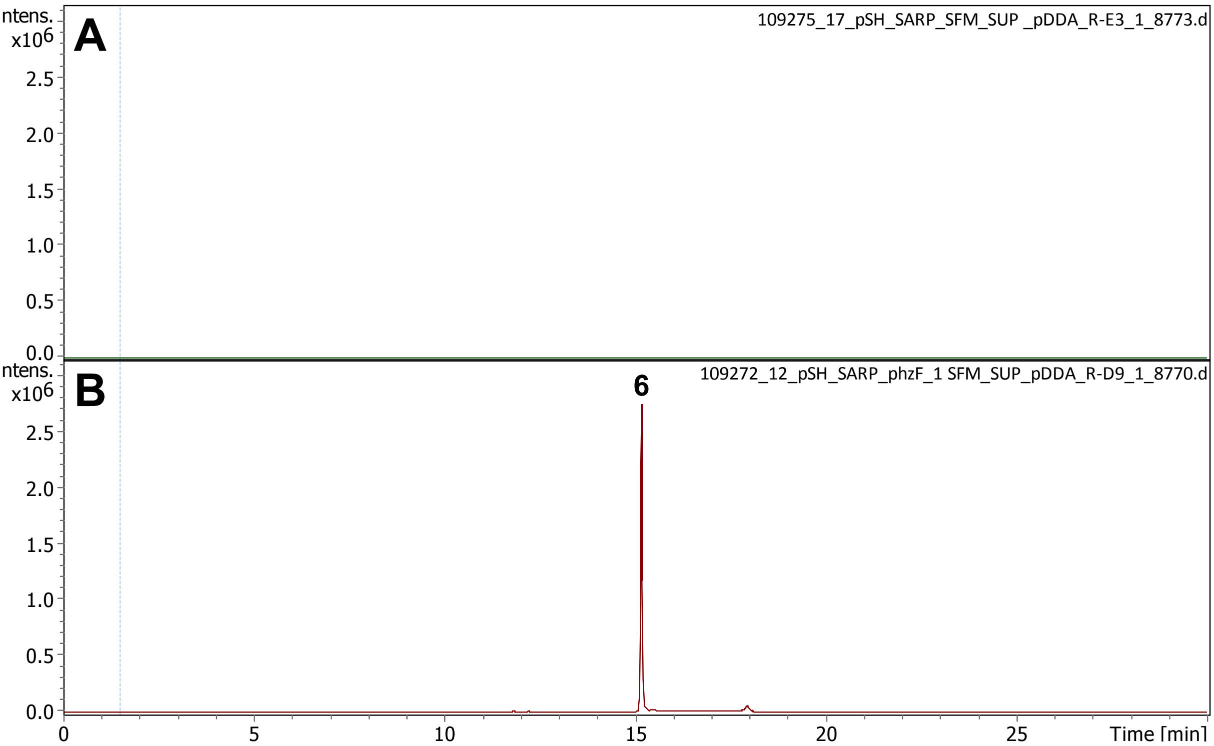

Figure S21. Extracted ion chromatograms (*m/z* 269.0557 ± 0.0050, corresponding to the $[M+H]^+$ ion of $C_{14}H_8N_2O_4$) obtained by LC-MS in positive ion mode of the methanol extracts from the supernatant of the *S. coelicolor* M1154 strains carrying the plasmids *pCLY10::mfq*/pOE_*mfqF* (A) and *pCLY10::mfq*/pOE_*phzF-mfqF* (B) grown in SFM medium. The peak corresponding to the tentatively identified phenazine-1,6-dicarboxylic acid (**6**) is labelled.

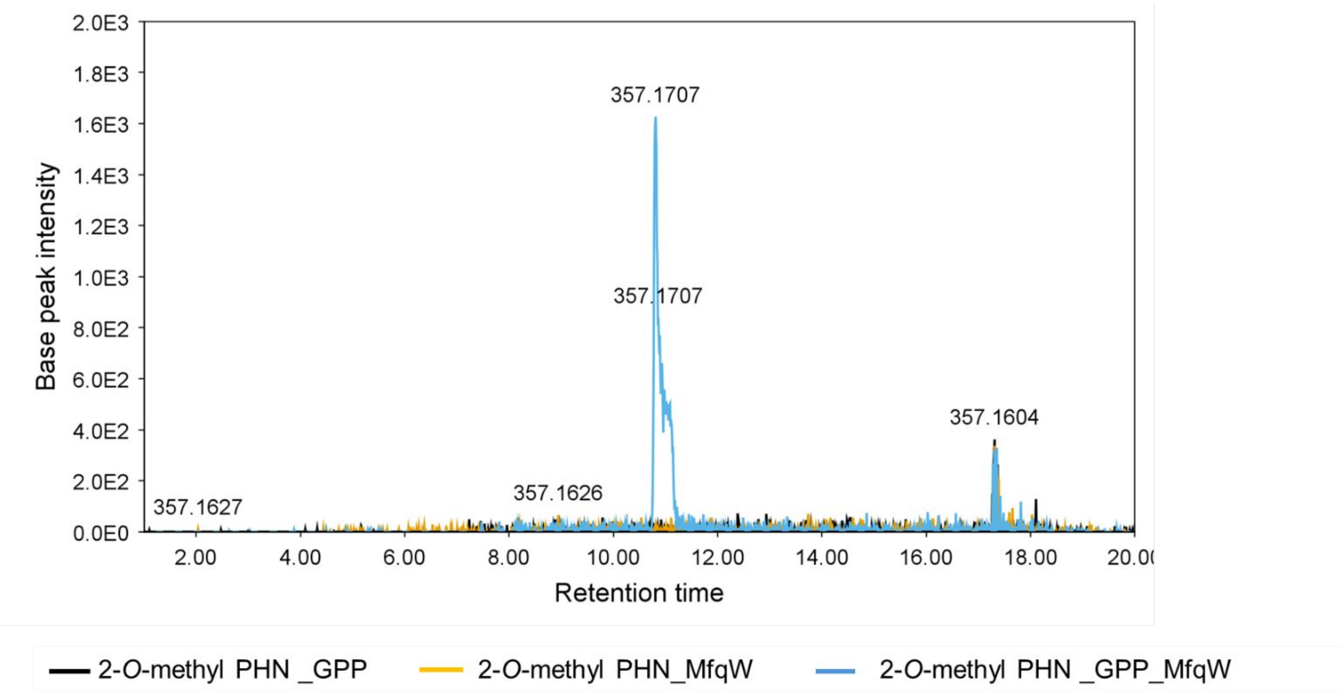

Figure S22. In vitro prenylation assay of MfqW with reduced 2-O-methylflaviolin (via dithionite, yielding 2-*O*-methyl PHN). LC-MS analysis was performed in negative ion mode. No prenylated products were detected in control samples containing either 2-*O*-methyl PHN + GPP (black line) or 2-O-methylflaviolin + MfqW alone (yellow line). In the full reaction containing 2-O-methyl PHN, GPP, and MfqW (blue line), an extracted ion chromatogram peak corresponding to the geranylated product was observed.

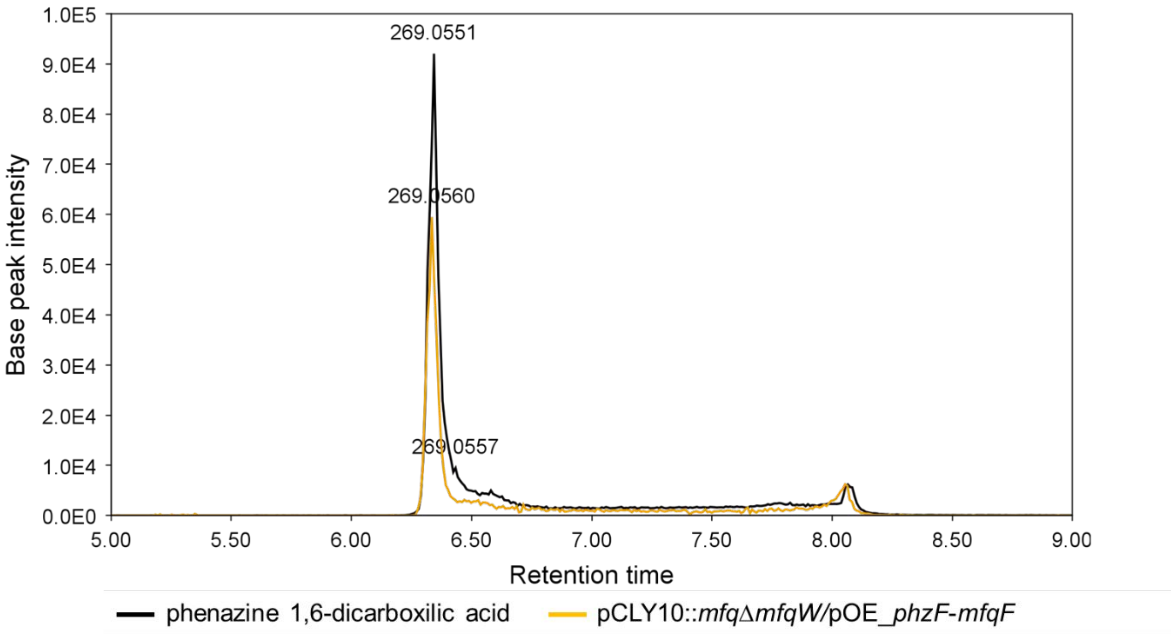

Figure S23. Extracted ion chromatogram of m/z 269.0557 ± 0.0050, corresponding to the $[M+H]^+$ ion of $C_{14}H_8N_2O_4$, obtained by LC–MS in positive ion mode from the methanol extract

of the culture supernatant of *Streptomyces coelicolor* M1154 carrying plasmids pCLY10::*mfq*Δ*mfq*W/pOE_*phzF-mfqF* grown in SFM medium. The peak corresponds to the tentatively identified phenazine-1,6-dicarboxylic acid (PDC).

References:

(1) Flett, F.; Mersinias, V.; Smith, C. P. High efficiency intergeneric conjugal transfer of plasmid DNA from Escherichia coli to methyl DNA-restricting streptomycetes. *FEMS Microbiol. Lett.* **1997**, *155*, 223–229.

(2) Brachmann, C. B.; Davies, A.; Cost, G. J.; Caputo, E.; Li, J.; Hieter, P.; Boeke, J. D. Designer deletion strains derived from Saccharomyces cerevisiae S288C: A useful set of strains and plasmids for PCR-mediated gene disruption and other applications. *Yeast* **1998**, *14*, 115–132.

(3) Jakočiūnas, T.; Bonde, I.; Herrgård, M.; Harrison, S. J.; Kristensen, M.; Pedersen, L. E.; Jensen, M. K.; Keasling, J. D. Multiplex metabolic pathway engineering using CRISPR/Cas9 in Saccharomyces cerevisiae. *Metab. Eng.* **2015**, *28*, 213–222.

(4) Gómez-Escribano, J. P.; Bibb, M. J. Engineering Streptomyces coelicolor for heterologous expression of secondary metabolite gene clusters. *Microb. Biotechnol.* **2011**, *4*, 207–215.

(5) Myronovskyi, M.; Rosenkranzer, B.; Nadmid, S.; Pujic, P.; Normand, P.; Luzhetskyy, A. Generation of a cluster-free Streptomyces albus chassis strain for improved heterologous expression of secondary metabolite clusters. *Metab. Eng.* **2018**, *49*, 316–324.

(6) Luzhetskyy, A.; Zhu, L.; Gibson, M.; Fedoryshyn, M.; Dürr, C.; Hofmann, C.; Hoffmeister, D.; Ostash, B.; Mattingly, C.; Adams, V.; Fedorenko, V.; Rohr, J.; Bechthold, A. Generation of novel landomycins M and O through targeted gene disruption. *ChemBioChem* **2005**, *6*, 675–678.

(7) Bilyk, O.; Sekurova, O. N.; Zotchev, S. B.; Luzhetskyy, A. Cloning and heterologous expression of the grecocycline biosynthetic gene cluster. *PLoS One* **2016**, *11*, e0158682.

(8) Chen, K.-Y.; Wang, H.-Q.; Yuan, Y.; Mou, S.-B.; Xiang, Z. Chemoenzymatic synthesis of cylindrocyclophanes A and F and merocyclophanes A and D. *Angew. Chem. Int. Ed.* **2023**, *62*, e202307602.

(9) Vagstad, A. L.; Hill, E. A.; Labonte, J. W.; Townsend, C. A. Characterization of a fungal thioesterase having Claisen cyclase and deacetylase activities in melanin biosynthesis. *Chem. Biol.* **2012**, *19*, 1525–1534.

(10) Pittelkow, M.; Boas, U.; Christensen, J. B. Carbocations in action: Design, synthesis, and evaluation of a highly acid-sensitive naphthalene-based backbone amide linker for solid-phase synthesis. *Org. Lett.* **2006**, *8*, 5817–5820.

(11) Husain, S. M.; Schätzle, M. A.; Röhr, C.; Lüdeke, S.; Müller, M. Biomimetic asymmetric synthesis of (R)-GTRI-02 and (3S,4R)-3,4-dihydroxy-3,4-dihydronaphthalen-1(2H)-ones. *Org. Lett.* **2012**, *14*, 3600–3603.

(12) Bell, A. A.; Stipanovic, R. D.; Puhalla, J. E. Pentaketide metabolites of Verticillium dahliae: Identification of (+)-scytalone as a natural precursor to melanin. *Tetrahedron* **1976**, *32*, 1353–1356.

(13) Wang, W.; Xue, J.; Tian, T.; Zhang, J.; Wei, L.; Shao, J.; Xie, Z.; Li, Y. Total synthesis of (±)-δ-rubromycin. *Org. Lett.* **2013**, *15*, 2402–2405.
